# Effectiveness of pedometer-based walking programmes in improving some modifiable risk factors of stroke among community-dwelling older adults: a systematic review, theoretical synthesis and meta-analysis

**DOI:** 10.1186/s12877-024-05069-z

**Published:** 2024-06-13

**Authors:** Sam Chidi Ibeneme, Juliet Mah, Chidimma Omeje, Gerhard Fortwengel, Akachukwu Omumuagwula Nwosu, Frank Onyemaechi Irem, Georgian Chiaka Ibeneme, Hellen Myezwa, Martins Nweke

**Affiliations:** 1 Department of Medical Rehabilitation, Faculty of Health Sciences, College of Medicine, University of Nigeria, Enugu Campus, Enugu, Enugu State, Enugu, Nigeria; 2 Faculty III, Hochschule Hannover University of Applied Sciences & Arts, 30159 Hannover, Lower Saxony Germany; 3 Department of Physiotherapy, University of Nigeria Teaching Hospital, Ituku/Ozalla, Enugu, Enugu State, Enugu, Nigeria; 4 Department of Nursing Sciences, Faculty of Health Sciences & Technology, College of Health Sciences, Ebonyi State University, Ebonyi State, Abakaliki, Nigeria; 5 Department of Physiotherapy, Faculty of Health Sciences, School of Therapeutic Studies,University of the Witwatersrand, 7 York Road, Parktown, Johannesburg, 2193 Gauteng South Africa; 6 Department of Physiotherapy, Faculty of Health Sciences & Technology, David Umahi Federal University of Health Sciences, Uburu, Ebonyi State Nigeria; 7 Department of physiotherapy, Faculty of Health Sciences, University of Pretoria, Pretoria, South Africa; 8 Department of Nursing Science, Faculty of Health Sciences & Technology, David Umahi Federal University of Health Sciences, Uburu, Ebonyi State Nigeria

**Keywords:** Community-dwelling older adults, Stroke prevention, Modifiable risk factors, Pedometer-based walking

## Abstract

**Background:**

Pedometer-based walking programs hold promise as a health promotion strategy for stroke prevention in community-dwelling older adults, particularly when targeted at physical activity-related modifiable risk factors. The question arises: What is the effectiveness of pedometer-based walking program interventions in improving modifiable stroke risk factors among community-dwelling older adults?

**Method:**

Eight databases were searched up to December 2^nd^, 2023, following the Preferred Reporting Items for Systematic Review and Meta-Analysis protocol. Inclusion criteria focused on randomized controlled trials (RCTS) involving community-dwelling older adults and reported in English. Two independent reviewers utilized Physiotherapy Evidence Database (PEDro) tool to extract data, assess eligibility, evaluate study quality, and identify potential bias. Standardized mean difference (SMD) was employed as summary statistics for primary —physical activity level —and secondary outcomes related to cardiovascular function (blood pressure) and metabolic syndrome, including obesity (measured by body mass index and waist circumference), fasting blood sugar, glycated hemoglobin, high-density lipoprotein cholesterol (HDL-C), and triglycerides. A random-effects model was used to generate summary estimates of effects.

**Results:**

The review analyzed eight studies involving 1546 participants aged 60-85 years, with 1348 successfully completing the studies. Across these studies, pedometer-based walking programs were implemented 2-3 times per week, with sessions lasting 40-60 minutes, over a duration of 4-26 weeks. The risk of bias varied from high to moderate. Our narrative synthesis revealed positive trends in HDL-C levels, fasting blood sugar, and glycated hemoglobin, suggesting improved glycemic control and long-term blood sugar management. However, the impact on triglycerides was only marginal. Primary meta-analysis demonstrated significantly improved physical activity behavior (SMD=0.44,95%CI:0.26, 0.61,*p*=<0.00001;I^2^=0%;4 studies; 532 participants) and systolic blood pressure (SMD=-0.34,95%CI:-0.59,-0.09;*p*=<0.008;I^2^=65%,2 studies;249 participants), unlike diastolic blood pressure (SMD=0.13,95%CI:-0.13,-0.38,*p*=0.33; I^2^=91%; 2 studies; 237 participants). Interventions based on social cognitive, self-efficacy, and self-efficiency theory(ies), and social cognitive theory applied in an ecological framework, were linked to successful physical activity behavior outcomes.

**Conclusion:**

Pedometer-based walking programs, utilizing interpersonal health behavior theory/ecological framework, enhance physical activity behavior and have antihypertensive effects in community-dwelling older adults. While they do not significantly affect diastolic blood pressure, these programs potentially serve as a primary stroke prevention strategy aligning with global health goals.

**Trial registration:**

Registration Number: INPLASY202230118

**Supplementary Information:**

The online version contains supplementary material available at 10.1186/s12877-024-05069-z.

## Introduction

### Background of the study

Stroke is a non-communicable disease that significantly impacts older adults, with incidence rates doubling after the age of 55 [1, 2]. It can result from either acute hemorrhage due to increased intravascular pressure or insidious necrotic changes in brain tissues caused by cerebral artery occlusion (infarct) [3]. The pathological sequelae of stroke include gross neurological deficits and motor disabilities due to compromised cortical inhibition of the lower motor system [4]. As a consequence, primitive reflexes [5] become more pronounced, interfering with functional limb movements. Upper [6] and lower [7] limbs may exhibit predominant extensor and flexor deformities, respectively. These stroke-related disabilities significantly limit functional independence in activities of daily living, social participation, productivity, and economic self-sufficiency, often leading to mood disorders [8–11] and a diminished quality of life [12]. Given the multifaceted impact of stroke, prevention strategies are of utmost importance. These strategies focus on modifiable risk factors related to physical activity, including physical inactivity, obesity, high blood pressure, and poor metabolic outcomes such as hyperglycemia (diabetes) and unfavorable lipid profiles [13]. Therefore, promoting a physically active lifestyle among community-dwelling older adults using pedometer-based walking programs emerges as a relevant and prioritized stroke prevention strategy in public health.

The World Health Organization (WHO) emphasizes the critical role of physical activity in promoting overall health. Specifically, adults should engage in a minimum of 150 minutes of moderate-intensity aerobic physical activity weekly to enhance cardiorespiratory and muscular fitness and reduce the risk of non-communicable diseases, including stroke [14]. However, despite these recommendations, community-dwelling older adults often fall short of meeting this requirement [15]. Fortunately, walking emerges as the most common type of physical exercise among this population [16]. It is easily incorporated into daily routines and contributes significantly to health promotion, particularly by reducing stroke risk factors associated with physical inactivity [15]. Epidemiological data reveal that low physical activity levels significantly contribute to the global disease burden. In 2016 alone, approximately 1.4 million deaths were attributed to physical inactivity—an 18.4 % increase since 2006 [17]. This trend is particularly pronounced in Lower-middle-income countries (LMICs), where physical inactivity accounts for 75 % of disability-adjusted life years (equivalent to 10.1 million disability-adjusted life years) lost due to non-communicable diseases, including stroke [18]. The economic implications are substantial, with physical inactivity projected to cost global healthcare systems approximately $27 billion annually between 2020 and 2030, reaching an estimated total of about $300 billion by 2030 [19]. Surprisingly, despite this knowledge, a significant portion of adults—27 %—remain physically inactive in 2022, failing to meet the WHO’s recommended physical activity threshold for optimum health [20]. Hence, promoting physical activity remains a critical global health priority. Interventions such as device-assisted walking programs, including pedometer-based interventions, can play a pivotal role in addressing this challenge. These programs should align with the World Health Organization’s global action plan on physical activity (2018-2030) [21] and contribute to Sustainable Development Goal (SDG) Target 3.4, which aims to reduce the burden of non-communicable diseases and enhance population well-being.

Physical activity data from 22 African countries, subjected to meta-analysis, reveal that 16v% of males and 24 % of females are inactive, similar to developed countries [22]. Specifically, in Nigeria, the most populous African nation, 25–57 % of the population is physically inactive [23, 24], with females being more likely to be inactive [23, 25]. This finding aligns with an earlier observation that females in a Nigerian population were more prone to stroke than males [11]. Additionally, this observation may have regional significance for Africa, where stroke prevalence is 1,460 cases per 100,000 person-years, while incidence is 316 cases per 100,000 person-years [26]. Therefore, strategies promoting physical activity, such as device-assisted walking programs, are necessary and have been proven effective in older adults [27]. Device-assisted walking programs, including pedometer-based interventions, could particularly benefit high-risk populations, including community-dwelling older adults [13]. Therefore, advocating for physical activity through programs like pedometer-based walking holds promise for improving health outcomes and preventing strokes in African communities.

Walking is a popular, familiar, suitable, effective, and cost-free form of physical activity for older adults [28]. It seamlessly integrates into daily activities and can be sustained into old age [29], thereby significantly improving health outcomes [30]. By reducing physical inactivity, walking plays a crucial role in mitigating cardiovascular diseases (such as hypertension), metabolic syndrome (including obesity) [31, 32], diabetes, and other physical inactivity-related conditions linked to stroke [33, 34]. Despite the natural decline in participation in walking and other physical activities with aging [35], mainly due to age-related changes in the locomotor apparatus [36], it remains crucial to emphasize stroke prevention strategies. For instance, promoting regular walking habits among community-dwelling older adults using pedometers is advised, with an emphasis on brisk walking [37]. While some authors [26, 38, 39] suggest that pedometer-based walking programs can help reduce cardiovascular diseases and metabolic syndrome in older adults, others [40] have found no significant improvement. The question that arises is: What is the effectiveness of a pedometer-based walking intervention in improving modifiable risk factors for stroke, including physical inactivity (measured by physical activity measures/metrics such as physical activity level-PAL, step count, distance traveled, calories burned, time spent in different intensity zones), components of metabolic syndrome (such as obesity measured by body mass index-BMI and waist circumference), elevated blood sugar (measured by fasting blood sugar and glycated hemoglobin), and abnormal triglycerides/HDL-C, as well as cardiovascular parameters (specifically high blood pressure) among community-dwelling older adults?

## Methods

### Research design

This systematic review examined randomized controlled trials on the effectiveness of a pedometer-based walking program in modifying stroke risk variables among older community residents. The study was registered on March 23, 2022, on the International Platform of Registered Systematic Reviews and Meta-Analysis Protocols (INPLASY)—registration number: INPLASY202230118.

### Eligibility criteria

When selecting studies for this review, the following eligibility criteria were considered:

A *Inclusion Criteria:*
  i. *Types of Studies:* This study reviews RCTs assessing the impact of pedometer-based walking programs on stroke risk factors.
*Stroke Risk Factors:* These include physical inactivity (measured by step count, distance traveled, calories burned, time spent in different intensity zones, physical activity level-PAL) and components of metabolic syndrome (such as blood pressure, obesity measured by body mass index-BMI and waist circumference, diabetes measured by fasting blood sugar and glycated hemoglobin, and high triglycerides/HDL-C).
    The studies included were published in English-language conference proceedings and peer-reviewed journals.
  ii. *Types of Participants:*
    This review included studies on exercise interventions using pedometer-based walking programs involving community-dwelling older adults aged ≥ 60, regardless of gender, who had modifiable risk factors for stroke, without specific limitations on the study setting.
    i. *Intervention:*
      We selected RCTs focusing on pedometer-based walking interventions for older adults, with a specific emphasis on supervised programs. These programs had no limitations regarding intervention dosage, form, frequency, duration, intensity, or post-intervention follow-up time.
    ii. *Types of Control:*
      Our study involved community-based RCTs among older individuals. The control groups fell into the following categories:
      i. *No Intervention (No-Contact Control Group):* Participants received no specific interventions.
      ii. *Different Interventions (Active Control Group): *Participants received alternative interventions. Counseling, Phone Calls, Health Information, Pamphlets, Education Sessions, and Advice on Increasing Walking Time, Self-Selected Intensity Exercise Programs, or pedometer plus other interventions, and
      iii. *Social Support (Social Control Group):* Participants received various forms of support and guidance.
  iii. *Timing:*
    Only studies that completed outcome assessments after the intervention or at least six months after the intervention, were included.
  iv. *Types of Outcomes:*
    Studies were included if they measured changes in modifiable risk factors for stroke. The primary outcome was physical activity, while some parameters related to cardiovascular function and metabolic syndrome served as secondary outcomes. All studies focusing on modifiable risk factors for stroke were included, analyzed, and combined. Clinical outcomes were evaluated and ranked, preserving the initial descriptions in the texts.
    i. *Primary Outcomes:*
       - *Physical activity* *:*
      The primary outcome is physical activity which refers to any movement of the body produced by the skeletal muscles that requires the use of energy is considered physical activity. It includes a range of activities, such as recreational pursuits, travel-related movements, and job-related duties. Physical activity level is the daily amount of physical activity a person engages in, used to calculate energy expenditure [41]. This encompasses both the duration and intensity of daily activities. Wearable technologies [42, 43], such as GPS, are reliable tools for assessing physical activity in both clinical [44] and community settings [45]. Accelerometers [46] and pedometers [47] also measure physical activity using parameters such as G-forces (g), meters per second squared (m/s²), heart rate, and pedometer step count.
    ii. *Secondary Outcomes:* *Metabolic Syndrome* *:*
      Metabolic syndrome is characterized by high blood pressure, excess body fat, abnormal cholesterol or triglyceride levels, and hyperglycemia (elevated blood sugar). It significantly increases the risk of heart disease, stroke, and type-2 diabetes.
      a *Obesity:* Obesity is diagnosed when a person’s body weight exceeds what is considered healthy for their height using various approaches including:
         - *Body Mass Index:*
        Body Mass Index is calculated by dividing body weight (in kilograms) by height squared (in meters), indicating the distribution of body weight relative to height [48].
         - *Waist Circumference:*
        Waist circumference is measured around the umbilicus of the stomach using a flexible, inelastic tape measure (a single measurement). This measurement serves to estimate fat distribution and screen individuals for weight-related health issues [49], including obesity, diabetes, cardiovascular diseases, and cancer. For increased accuracy and consistency, the measurement is repeated two more times, and the average of the three measurements is calculated and used. In males, low risk is below 94 cm, high risk is 94-102 cm, and very high risk is 102 cm. However, for females, low risk is below 80 cm, high risk is between 80 to 88 cm, and very high risk is above 88 cm [50].
         - *Waist-hip ratio:*
        Waist-hip ratio (WHR) is calculated by dividing an individual’s waist circumference by their hip circumference. Specifically: the waist circumference is measured at the narrowest part of the waist. The hip circumference is measured at the widest part of the hips or buttocks. WHR measures abdominal fat distribution, with a higher WHR indicating a greater proportion of abdominal fat, which has been associated with increased health risks like cardiovascular disease and diabetes. Monitoring WHR offers insights into an individual's health and risk factors.
      b *Hyperglycemia (elevated* *blood sugar):*
        Hyperglycemia refers to elevated blood glucose levels (commonly known as blood sugar). In practical terms it defines the point when blood sugar levels exceed the normal range. Chronically elevated blood sugar can lead to diabetes, either due to insufficient insulin production or inefficient utilization. To detect diabetes, serum glycated hemoglobin, continuous glucose monitoring, and glucometer tests are commonly used [51]. Fasting blood sugar is expressed in mg/dL or mmol/L, while glycated hemoglobin is expressed as a percentage (DCCT unit) or as a number in mmol/mol (IFCC unit).
      c *Lipid Profile:* A lipid profile, also known as a lipid panel, is a blood test that assesses blood lipid levels. Lipids are fats that cannot dissolve in blood. The key components measured in a lipid profile include:
         - *Total Cholesterol:* The sum of all cholesterol in your blood.
         - *Low-Density Lipoprotein (LDL) Cholesterol*: Often referred to as “bad” cholesterol, as high levels are associated with an increased risk of heart disease.
         - *High-Density Lipoprotein (HDL) Cholesterol: *Known as “good” cholesterol, as higher levels are beneficial for heart health.
         - *Triglycerides:* A type of fat found in the blood.
        Monitoring lipid levels through a lipid profile helps assess cardiovascular risk and guides preventive measures. This laboratory blood test measures triglycerides (mmol/L) and cholesterol (mg/dL or mmol/L) blood concentration using spectrophotometers and lipid panels [52].
      d *Cardiovascular Function (Blood pressure):*
        Cardiovascular function refers to the intricate workings of the heart and blood vessels within the body. The cardiovascular system regulates body temperature and adapts to stress, but various diseases can lead to heart attacks, strokes, arrhythmias, heart failure, and heart valve complications. Common detection methods include masks, ECGs, or EKGs [53], sphygmomanometers, pulse oximeters, Holter monitoring [54], echocardiograms, exercise tests [55], cardiac catheterization [56], heart (cardiac) computed tomography scan [57], and magnetic resonance imaging [58]. In the context of this study, the outcome measure of cardiovascular function specifically focuses on blood pressure. Blood pressure represents the force exerted by circulating blood against the inner walls of blood vessels. It is quantified in millimeters of mercury (mm Hg). This measurement provides valuable insights into cardiovascular health and function. Healthcare professionals utilize blood pressure measurements to assess cardiovascular health and monitor conditions such as hypertension. Blood pressure is divided into two determinations:
         - *Systolic Pressure:* Represents the maximum blood pressure during heart contraction.
         - *Diastolic Pressure:* Reflects the minimum pressure recorded before the next contraction when the heart relaxes between beats.
B *Exclusion Criteria:*
  1. Studies without intervention programs based on pedometers.
  2. Studies that used pedometer-based walking intervention but failed to assess the study’s primary objectives.
  3. Publications, opinion papers, narrative review syntheses, systematic reviews, and any correspondence without a clear methodology or main data description.
  4. In multiple publications from the same research project, the most recent publication on the subject was included.

### Information sources

This review employed a comprehensive search strategy, which included:

a *Hand Searches* *of Grey Literature*: The relevant information was meticulously searched beyond traditional academic sources, ensuring a thorough exploration of the literature landscape.
b *Screening Bibliographic Databases*: The databases were systematically examined to identify relevant studies. The search covered key databases such as AMED Trial registrations, CINAHL, EMBASE, PubMed, the Cochrane Library, and a directory of open-access repository websites (http://www.clinicaltrial).
c *Reference Lists of Included Citations Using the Snowballing Method*: The guidelines from the Cochrane Handbook of Systematic Reviews of Interventions [59] and the Centre for Reviews and Dissemination’s guidance for Health Care Review [60]. By tracing references within relevant articles, the search network was expanded.

i. *Search Strategy:*
  A study plan was developed using Medical Subject Heading (MeSH) search terms and keywords extracted from titles, abstracts, and text. A pilot test assessed sensitivity and specificity. The search instructions included truncators and Boolean operators. For PubMed, this review adapted a search technique outlined in Appendix I, adjusting title and syntax to accommodate other databases. The searches were conducted from inception until December 2, 2023. Additional resources were consulted beyond electronic database searches: Published Systematic Reviews of Exercise Interventions, Reference Lists of Pertinent Books and Articles, he Cochrane Systematic Review Database, The National Institute of Health Research Portfolio for recently concluded or ongoing studies, Identified studies and recommended papers’ reference lists, and The Current Controlled Trials Register.

### Study Record and Data Management

- *Search Results and Deduplication:* After exporting the search results into the RefWorks^TM^ manager, the records were meticulously deduplicated. Bibliographic entries were subsequently exported into Microsoft Excel [61] for easier organization and classification based on the particular inclusion and exclusion criteria. The review questions was also organized and improved (if necessary) to facilitate the sorting of articles, taking into account the inclusion and exclusion criteria.
- *Selection Process:* The screening process involved two reviewers: J.U. (Reviewer 1) performed an initial screening on the title and abstract. C.O. (Reviewer 2) separately cross-checked the results of the initial screening. Thereafter, both reviewers independently went through the full texts of each of the selected studies, applying the qualifying criteria for additional screening. Disagreements over whether an article should be included or excluded were resolved through discussions and reflections, with P.S.I. (reviewer 3) consulting as needed. In cases where a decision could not be made using the available information, study authors were contacted (up to three emails maximum) for clarification to address any questions regarding the selection of any given study.

### Data Collection Processes

1. *Risks of Bias Assessment in Individual Studies*
  The Physiotherapy Evidence Database (PEDro) 11-item scale was employed to rigorously evaluate the methodological quality of the selected studies [62]. The first item pertained to external validity, while the remaining 10 items assessed the internal validity of individual clinical trials. Notably, the study’s overall quality improved with higher scores on this scale, which was interpreted as follows: 9-10: Excellent, 6-8: Good/High, 4-5: Moderate/fair, <4: Poor
  Additionally, the quality of RCTs was assessed by assigning a score of “1” for each “yes” response and “0” for “no,” “unclear,” or “not applicable” (N/A) responses. Summarizing these scores using a critical appraisal tool allowed for the determination of the total number of “yes” responses out of 10. The evaluation process was conducted independently by reviewers 1 and 2. Furthermore, the study’s level of evidence was assessed based on both the sample size and the PEDro score [63, 64].

### Data collection processes

i. *Data Item:*
  Data from the included studies were meticulously extracted using a standardized data extraction form. The form encompassed various essential elements, including: Author’s reference, participant characteristics, inclusion/exclusion criteria, study sample details, intervention components, setting information, intervention delivery personnel, duration of the intervention and follow-up (if available), attrition rate, outcome assessment/measurement methods, results, conclusions, and funding sources
ii. *Data Synthesis and Assessment of Heterogeneity*
  In this review, the impact of a pedometer-based walking program on modifiable risk factors for stroke among older community residents, was investigated. To evaluate the intervention’s efficacy, a proof table was constructed and quantitative results were analyzed. The following steps were taken:
*Statistical Methodology:*
  This review adhered to the standard Cochrane meta-analyses procedure. For each variable, an appropriate statistical method was applied.
  1. *Risk Ratio (RR): *For dichotomous variables, the risk ratio along with a 95% confidence interval (CI), were calculated.
  2. *Weighted Mean Difference (WMD): *For similar outcomes, the post-intervention weighted mean difference, was computed.
  3. *Standardized Mean Difference (SMD): *For varied measures, this review determined the SMD. Interpretation of the SMD values followed Schünemann et al.'s [65] recommendations: Big values: exceeding 0.70, Moderate values: between 0.40 and 0.70, Small values: between 0.00 and 0.39

### Data analysis

The analysis of studies involved a comprehensive examination of various factors, including: Year of publication, Author references, Sample size, Age distribution, Study settings, Data collection format, Outcome measures, Intervention and control components, Format of intervention delivery, Intervention and follow-up durations

i. *Narrative Synthesis.* To explore relationships and draw conclusions from diverse studies, this review followed the Centre for Reviews and Dissemination’s narrative synthesis guidelines. By focusing on primary outcomes, we conducted an investigation and presented our findings.
ii. *Meta-Analyses: *Three meta-analyses were performed using a random-effects model to determine pooled effect sizes across the trials according to the Cochrane Handbook for Systematic Reviews of Interventions guidelines [60]. Heterogeneity values were categorized as follows: Low: 25 %, Medium: > 25 % – 75 % and High: > 75 %

The heterogeneity was assessed using the Higgins I2 test and Cochrane’s χ2 test (with a 10 % significance threshold). This review focused on heterogeneous studies, employing narrative synthesis to elucidate relationships and findings both within and between the research studies, consistent with the Centre for Reviews and Dissemination’s recommendation [66].

### Sensitivity analysis

To explore the potential impact of significant heterogeneity—arising from different intervention types or comparators—a sensitivity analysis was conducted. This assessment considered bias impact in high-risk studies. Additionally, subgroup analyses investigated heterogeneity in treatment effects, involving more than two studies with comparable subsets.

### Rating quality of evidence and strength of recommendation

The systematic review’s recommendation strength underwent rigorous assessment using two key tools:

1. *Physiotherapy Evidence Database Scale (PEDro):* This scale [67], considered the internal validity of the research. Additionally, statistical reporting played a crucial role in shaping the review’s decision.
2. *Verhagen’s Delphi List:* Verhagen’s list [68], evaluated the methodological quality of studies, categorizing them as follows: High Quality (low risk of bias), Moderate Quality and Low Quality (high risk of bias).

### Evidence statement and quality assessment

Each evidence statement was meticulously rated based on its quality: *High Quality*: Implies that additional research is unlikely to alter the effect estimates.; *Moderate Quality* ***:*** Suggests that further research could significantly impact the effect estimates. *Low Quality* ***:*** Indicates that additional research is very likely to alter or significantly change the estimate. The assessments were based on the PEDro score**,** which reflects methodological quality as follows: 9–10**:** Outstanding, 6–8: Good, 4–5**:** Fair, and 4–6**:** Poor

### Level of evidence

Study’s level of evidence was determined by both the sample size and the PEDro score:

- *Level 1* Evidence (Good or Outstanding): High-quality randomized controlled trials (RCTs) with larger samples.
- *Level 2* Evidence: Lower quality rating (fair or poor) and a sample size less than 50. [62, 63].

### Report of review

This systematic review adhered to the PRISMA statement requirements [69]. The final report included relevant material and a PRISMA checklist (Appendix II).

## Results

### Study selection

The study initially analyzed 425 citations from included sources. After removing 179 duplicates**,** titles and abstracts of remaining 246 papers were screened, resulting in 36 publications. Following full-text screening, eight articles [70–77] met the inclusion criteria.

### Reasons for exclusion

**Fig. 1:**
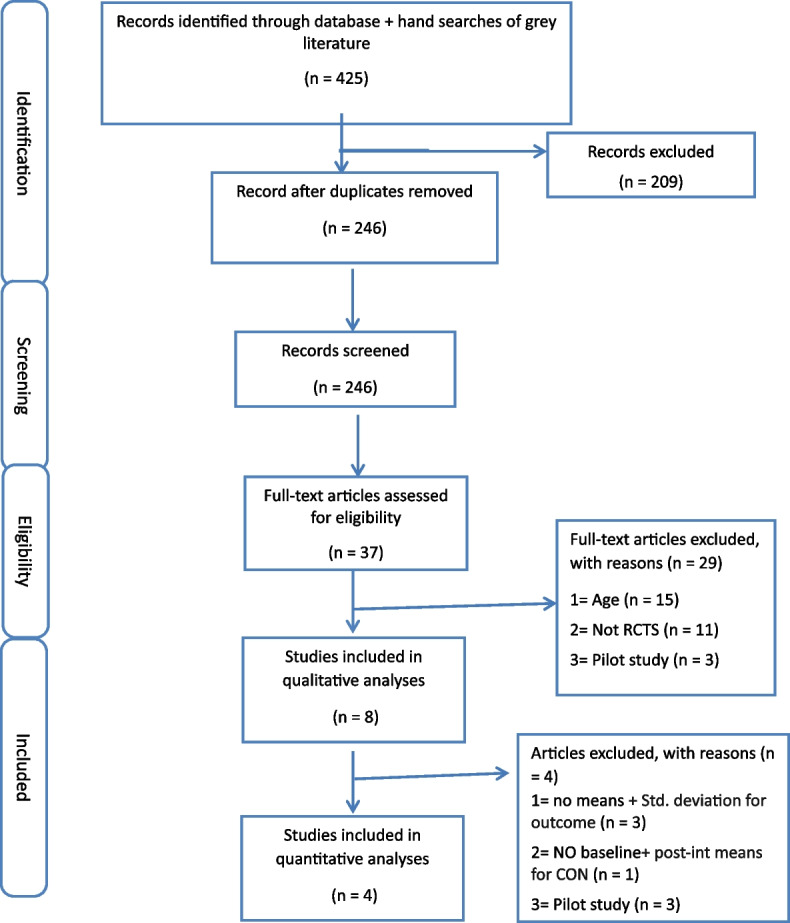
PRISMA flow diagram for modifiable risk factors of stroke; *adapted from Moher, Shamseer &* Clarke [102] Preferred Reporting Items for Systematic Reviews and Meta-Analyses

In this review, 14 full texts were excluded because they involved participants outside the required age range of the study [41, 78–90]. Additionally, eleven papers [91–101] were excluded due to non-randomized controlled trial designs. Furthermore, three citations represented pilot studies, and their details are provided in the flowchart (Fig. 1) below.

- *Qualitative Study: *After excluding the aforementioned papers, eight studies [70–77] were included in the qualitative study.
- *Quantitative Study:* Out of the eight papers [70–77] included for qualitative analysis, four papers [72, 74–76] were excluded from quantitative or meta-analysis for the following reasons: one study [72]: did not report standard deviations for fasting blood sugar and diastolic blood pressure, one study [74]: lacked information on changes in fasting blood sugar, BMI, physical activity, systolic, and diastolic blood pressure in standard deviations. Two studies [72, 75]: did not provide post-intervention means and standard deviations for physical activity variables, walking frequency, diastolic, and systolic blood pressures, and one study [76]: did not furnish baseline and post-intervention mean of physical activity (steps) for the control group.

### Included studies

Eight studies [70–77] were included in this review.

i. *Participants of Included Studies:*
  This review involved 1546 older adults, including 691 males and 848 females - excluding seven participants from one study [77] because their gender was not accounted for. Participants were aged ≥ 60 and were either physically inactive, living a sedentary lifestyle, or had modifiable risk factors for stroke. Studies were conducted in different countries: one study each in USA [73] Norway [72], Japan [76], China [77], Australia [70], New Zealand [74], Taiwan [75], and Scotland [71], with varying results across different countries.
ii. *Intervention:*
  This review included eight RCTs [70–77] (Table 1). These studies examined modifiable risk factors for stroke, including:
  - *Physical activity:* Measured by physical activity level, accelerometer counts, self-reported level of walking frequency, physical activity daily minutes, total walking activity (minutes per week), and daily step counts.
  - *Metabolic* *syndrome*: Assessed via obesity indicators (body mass index, waist-hip circumference), fasting blood sugar, glycated hemoglobin, and lipid profile (triglycerides, low-density lipoproteins, and HDL).
  - *Cardiovascular function*: Specifically systolic and diastolic blood pressure were assessed.

**Table 1:** Study characteristics

**Study, (Country), Design, Quality, Attrition,**	**Participants** **Disease severity/status** **Age range(mean)** **Sample size,** **Retention (attrition)** **Gender**	**Description of Interventions** **Gender** **Mean age** **No. randomized (on completion)** **Disease history** **Duration** **Theoretical framework**	**Description of Control** **Gender** **Mean age** **No. randomized (on completion)** **Disease history**	**Outcomes; When it was accessed**	**Outcome measures**	**Summary of Results**
Bjorgaas et al. [2008] (Norway), RCT, 31.4% [72]	Older T2DM adults NR < 80 ( 56.4 ± 11.0) *N* = 70 *n*=48 (*n* = 22). Men=45; women= 25	G_1;_ Recorded pedometer steps 3 weekdays, twice/mth. X 6 mths. increase dly. step count from one visit to the next. NR 56.4 ± 11.0 yrs 33 (23) DX hx: 4.6 ± 5.20 yrs 6 mths NR	No pedometer + advice to increase Ave. dly time spent walking from one visit to another, guided by the logbook. NR 61.2 ± 9.70 yrs 37 (25) DX hx: 7.4 ± 5.40 yrs	Steps/day SBP DBP Weight BMI WHR FPG HBA1c Total Cholesterol, HDL cholesterol, Triglycerides. VO_2_peak (L/min) VO2peak (mL/[kg min]) @Baseline, 1-mnt, 3-mnts & 6-mnts	SBP = NR DBP = DBP Weight + NR BMI = NR WHR = NR FPG, HbA1c, & by std. mth lipids; Vo_2peak_ = mask (Metamax II; Cortex, Leipzig, Germany);	No diff. in steps per day(*P* = .65) No diff. in Weight, HbA1c, FPG, TGC, DBP, & HDL-C (*p*=>.38), Vo_2_peak did not increase from study month 1 to 6 (*p* > .17)
Furber et al. [2010] (Australia), RCT 6.5% [70]	Older adults with Cardiac dxs 59 (26.57 %) poor status NR (66.7 ± 10.60) *n*=215 *(n = 204)* NR (NR) males = 151; females = 64	G_1:_ Self-monitoring of PA using a pedometer (Yamax Digiwalker 700B) + step calendar; + 15 mins behavioural counselling & goalsetting sessions via telephone support given 1 wk. after pedometer int. + received two PA info. brochures by mail. Received booster phone calls at 12 wks. & 18 wks. males = 75; females = 29 66.7 ± 10.60 yes 104 (97) 6 weeks Theory: Int. was developed based on Social cognitive theory focusing on enhancing self-efficacy, increasing beliefs about the positive health effects of taking action (outcome expectancies) & establishing PA goals	Received two PA info. Brochures by mail. 65.4 ± 11.50 yrs 111 (107) males = 76; females = 35	Self-reported PA per wk @Baseline, 6 wks & follow-up 6 mnts.	- The Active Australia Questionnaire for self-reported PA.	Sig. increase in total PA mins; total PA sessions; walking mins & walking sessions
Kerr et al. [2018] (USA), Cluster RCT 21.4% [73]	Older adults with no specific health conditions. NR ≥ 65 yrs (mean age= NR) *N* = 307 *n*=217 (n = 90) males=151; females = 64	G_1_: 4 individual telephone counselling x 8 weeks & self-monitoring + pedometers walking int, Grp education sessions, grp walks, community advocacy & pedestrian community change projects. Females = 71 % 85.3±6.5 years *Follow-up:* At 3 mths: 151 at baseline (128 at 3 mnts ) At 6 mths: 151 at baseline (124 at 6 mnts) Duration: 12 months *N*= 151 (completed=120, drop out= 31) Theory: Social Cognitive Theory techqs. were employed & applied in an Ecological framework with int. activities occurring at the individual (goalsetting), interpersonal (group walks), and community levels (pedestrian-advocated improvements in walkability).	G_0_ : Education sessions + health calls 81.9 ± 5.9 years Females = 74 % *Follow-up:* At 3 mths: 156 at baseline (137 at 3 mnts ) At 6 mths: 156 at baseline (127 at 6 mnts) *N*= 156 (completed= 121, drop out = 35)	*Primary outcome:* PA. *Secondary outcomes*: Blood pressure @Baseline, 3 mnts, 6 mnts., 9 mnts., & 12 mnts.	Accelerometer	There were interaction effects (*p* < .001), by age, marital status and education & gender which also had the greatest value. PA sig. increased in the G_1_ (56 min of MVPA per week; 119 min of light PA) compared with the G_0_ & remained sig. higher for 12 mnts. G_1_: Men almost doubled their PA levels from baseline G_0_: Men greatly decreased their PA levels. SBP sig. decreased (*p* < .007) & likewise DBP (*p* < .02) @ 6mnts.
Kolt et al.[2012] (New Zealand), RCT 18.2% [74]	Low-active older adults NR > 65 yrs (NR) *N*= 330 *N *= 270 (n = 60) Men= 152; women=178	G_1_: Pedometer-based walking + initial face-to-face advice on engaging in PA + 3 telephone counselling follow-up sessions (> 3 to 4mns) + moderate-intensity multi-component Exs or mind–body Exs; 45–60mins; 3 times/wk x >6mnths + 3 Telephone counselling x 12 wks comprising; Call 1 - (Information provision & goalsetting - 15-30mins) + Call 2 - (Assessing progress & further goalsetting - 10-15 mins), + Call 3 - providing further encouragement & discussions around relapse prevention - 10-15mins) 74.3±6.2 yrs No of participants: 165 (140 completed) Duration: 12 months Theory: NR	G_0_: Received same int. except that counselling focused on accumulating PA around time-related goals rather than step-related goals. 73.9 ± 5.9) No of participants: 165 (130 completed)	PA Blood pressure BMI @Baseline,3-mth& 12-mth (follow up)	Auckland Heart Study PA Questionnaire	At 12 months, minutes of leisure walking differed significantly between groups (*P* = .03) over time. Neither group had a change in BMI in this study. No sig. between grp change in BP
Lee et al. [2007] (Taiwan), RCT 8.9% [75]	Older adults with HTN NR Age range: aged 60 years and over (71.3±6.4) *N* = 202 *n*=184 (*n*= 18) Men= 118, women=84	G_1_: Ccommunity-based walking int., face-to-face & phone support to increase walking (pedometer + walking log provided; NO targeted value for PA mentioned) Pedometers: provided to motivate participants & facilitate walking. Duration: NR (individualised) Intensity: NR (individualised) Frequency: NR (individualised) No of participants: 102 (91 completed) Duration: 26 weeks (6 mnths) Theory: Self-efficacy theory constructs were applied in designing & measuring the outcome of the physical activity intervention. The assessment focused on self-efficacy expectations when using the pedometer as a motivator & thus evaluated participants’ degree of confidence to exercise in the face of barriers (bad weather & tiredness).	G_0_: Usual primary health care. No of participants: 100 (93 completed)	*Primary outcome:* DBP & SBP *Secondary outcome:* - Exercise self - efficacy score, - self-reported walking frequency, @Baseline & 26-wk.	BP measured with a traditional mercury sphygmomanometer	More participants in the G_1_ increased their regular walking (*p*<0.0005), exercise self-efficacy scores (1.23, 95% CI, 0.5 to 2.0, *p*= 0.001), self-reported walking (*p*< 0.0005) but not the DBP (*p*=0.19). Between baseline and six month follow-up, SBP sig. reduced in the G_1_ vs G_0_ (mean dif. = −7 mmHg (95% CI −11.5 to −2.5, *p*=0.002)
McMurdo et al [2010] (Scotland) Prospective RCT, 12% [71]	Sedentary older women aged 70 and older, (77.3±5.0) *N* = 204 *n*=174 (*n *= 30) NR	G_1_: BCI - comprising Brief education session focusing on beliefs & motivation for walking + a self-regulation theory-based int. was used for goalsetting, individualized activity action plans and plans to address barriers to action & coping, planning, self-monitoring, & feedback **PLUS** Moderate exercise PA training in sedentary older women. G_2_: BCI PLUS pedometer 68 (completed =53, drop out = 15) G_1_: 77.6±5.4 G_2_: 77.1±4.9 No of participants: 68 Duration: 6 months. Theory: The BCI was based on self-regulation theory, which emphasizes the role of goalsetting, planning, and self-monitoring behaviour change.	G_0_: Received the usual care. 77.0±4.9 yrs No of participants: 68 Completed= 66,, drop out= 2)	*Primary outcome:* Daily activity in mins @Baseline & 3 mnts	Accelerometry. (RT3 Accelerometry Research Tracker, Stay Healthy, Inc., Monrovia, CA). Omron HJ-113 piezoelectric pedometer (Omron Healthcare UK Ltd, Milton Keynes, UK)	At 3 mths: G_0_ had a decline in mins spent walking G_1 vs_ G_0_: Accelerometry counts sig. increased (*p* = .002) & also increased mins spent walking G_2_ vs G_0:_ Accelerometry counts sig. increased (*p* = .04) & had a small decrease in mins spent walking. At 6 mths: Accelerometry counts in both G_1_ & G_2_. decreased & were not sig. diff. from baseline & pedometer-based Int. provided no extra benefits in PA, but may have motivated participants to remain in the trial.
Yamada et al. [2012] (Japan) Pilot RCT 5.7% [76]	Sedentary older adults NR > 65 yrs (NR) *N* = 87 *N* = 82 (*n* = 5) Men= 47; Women = 40	G_1_: Pedometer-based behavioural change programs (no targeted value stated), consisted of motivation for walking + goal-setting, self-monitoring, & feedback No of participants: 43(40 completed) Duration: 6 months Theory: NR	G_0_: Received no intervention No of participants: 44 (42 completed)	Steps/day Walking time @Baseline & 6 mths	pedometer	Int. grp increased average dly steps by 83.4% (from 2031±1323 to 3726 + 1607) unlike G_0_. (2047 + 1698 to 2267 + 1837). Int. grp had Sig. & greater improvements in secondary outcome measures - LLM, walking time, TUG, & PA (P < 0.05).
Yang & Petrini [2018] (China) RCT [77]	Inactive retirees NR (64.32+5.395) *N* = 60 *n*=53 (*n* = 7) Male= 15; female= 38	INT: Pedometer-based walking with: (a) prescribed moderate-intensity, 3x/week, 24hrs interval between sessions; monitored by the RPE scale (b) Self-selected intensity (c) 50 min per session; (d) Aerobic walking or jogging; (e) volume was set as 5,400 - 7,900 steps/dy; (f) progression was an initial 30 min/session at the first wk & a 5- min increase in session time per wk over the first 4 wks until up to 50 min/session. No of participants: 30 (Completed= 26, drop out= 4) Duration: 6 months Theory: Hedonic theory provides a framework to explain how the pedometer-based physical activity intervention programme affected physical activity behaviour, while the dual-mode model was used to gain insight into the affective responses to exercise on varying intensities.	G_0_ : Self-selected intensity exercise No of participants: No of participants: 30 (Completed= 27,, drop out= 3)	Daily step counts Physical fitness indicators: - BMI - SBP & DBP - Daily Sep counts - waist Circumference - PA behaviour @Baseline, & 3 mnts (12 wks)	Pedometers - Stadio./Weighing Scale - Sphyg. - Tape measure Pedometer	Dly step counts, BMI, WC, PAL, DBP and SBP, did not differ sig. in G_1 vs_ G_0_. The former had a more +ve & less -ve affect on Exs.

*INT* Intervention, *G*_*0*_ Control, *PA* Physical activity, *BP* Blood pressure, *SBP* Systolic blood pressure, *DBP* Diastolic blood pressure, *WC* Waist circumference, *WHR* waist-hip-ratio, *Wk*. week, *HTN* hypertension, *MTh* month, *wk.* week, *dly* daily, *BCI* Behavioural change Intervention, *T2DM* type 2 diabetes mellitus, *dxs*. Diseases, *sphyg* sphygmomanometer, *sig.* significance, *TUG* Timed up and go test, Average ave, *hx*. History, *grp* group, *RCT* randomised controlled trials, moderate- vigorous Physical Activity *MVPA*, Negative *–ve*, Positive *+ve*, Exercises *Exs*, *PAL* Physical activity level, *RPE* The Borg’s Rating of Perceived Exertion, Leg muscle mass *LLM*

The average study duration was 6.175 ± 3.64 months. However, sessions were not consistently timed, except in only one study [75], which lasted between 30 minutes per session in first week and 50 minutes per session in fourth week and beyond. Participants received the intervention in a group setting in one study [71], and only one study [73] reported that the intervention was community-based. The summary of the interventions across the studies combined pedometer-based walking programs with other behavioral change strategies or interventions are presented below:

1. *McMurdo et al.* [71] – Behavioural Change Intervention (BCI) plus pedometer - comprising Brief education session focusing on beliefs & motivation for walking + a self-regulation theory-based int. was used for goalsetting, individualized activity action plans and plans to address barriers to action & coping, planning, self-monitoring, & feedback PLUS Moderate exercise PA training in sedentary older women + Pedometer
2. *Kerr et al.* [73] – Individual Counseling and Pedometer-Based Walking: Four individual telephone counselling sessions x 8 weeks & self-monitoring + pedometers walking int, group education sessions, group walks, community advocacy & pedestrian community change projects.
3. *Kolt et al.* [74] - Pedometer-Based Walking Intervention with Telephone Counseling: Pedometer-based walking + initial face-to-face advice on engaging in PA + 3 telephone counselling follow-up sessions (> 3 to 4mns) + Telephone counselling call 1 (Information provision & goalsetting - 15-30mins) + multi-component Exercises or mind–body Exercises; moderate intensity; 45–60mins; 3 times/wk x >6 mths + call 2 - (Assessing progress & further goal-setting - 10-15 mins), + call 3 - providing further encouragement & discussions around relapse prevention - 10-15mins).
4. *Lee et al.* [75] - Community-Based Walking Intervention PLUS pedometer: Community-based walking int., face-to-face & phone support to increase walking (pedometer + walking log provided; NO targeted value for PA mentioned).
5. *Yamada et al. *[76]* - Pedometer-Based Behavioral Change Programs:* Pedometer-based behavioural change programs (no targeted value stated), consisted of motivation for walking + goal-setting, self-monitoring, & feedback
6. *Furber et al.* [70] - Self-Monitoring of Physical Activity (PA) Using a Pedometer: Participants used a Yamax Digiwalker 700B pedometer and a step calendar + Received 15 minutes of behavioral counseling + goal-setting sessions via telephone support 1 week after starting the pedometer intervention + received two PA information brochures by mail + Booster phone calls given @ 12 weeks and 18 weeks.
7. *Bjorgaas et al.,* [72] - Pedometer-Based Walking Intervention: Recorded pedometer steps 3 weekdays, twice/month. X 6 months, and increased daily step counts.

However, one study used pedometer to monitor exercise intervention impact and is summarized below:

8. *Yang & Petrini* [77] - Self-Selected & Prescribed Intensity Exercise impact on physical activity monitored with pedometer: Pedometer used to monitor step counts after exposure to either (a) Prescribed moderate-intensity exercises: 3 times per week, 24-hour interval between sessions; Monitored by the RPE (Rate of Perceived Exertion) scale, OR (b) Self-selected intensity exercises, 50 minutes per session PLUS Aerobic walking or jogging, Volume set as 5,400 - 7,900 steps per day. Progression: Initial 30 minutes per session in first week, followed by a 5-minute increase in session time per week over first 4 weeks until reaching up to 50 minutes per session.

### Control groups sub-grouping

Among the eight studies [70–77] in our review, diverse control groups were utilized, and were sub-grouped in this review based on the types of control groups involved as shown below:

1. *No-Contact Control Group*: Three studies [70, 72, 77] were sub-grouped as the no-contact control group for receiving mainly usual care. In one study [77], it was not explicitly described as a separate group with a specific intervention. However, the study design involved comparing the effects of self-selected and prescribed intensity exercise with the control group, which likely received usual care or no specific exercise intervention.
2. *Active Control Group:* In two studies [71, 74] were sub-grouped as active control group for receiving different interventions or alternative therapies.
3. *Social Control Group:* Three trials [73, 75, 76] were sub-grouped as the social control group for receiving: phone calls, health information, health pamphlets, and social support.

### Outcomes

1. *Physical Activity Level*: Eight papers [70–77] examined the impact of pedometers on physical activity outcomes. Three (33.33 %) studies [74, 76, 77] assessed physical activity levels using step counts. In addition to this, some of the studies evaluated: Self-reported physical activity (including total physical activity minutes and total physical activity sessions) as well as walking variables (including walking minutes and walking sessions) [70], daily minutes of activity.[73], change in walking minutes over time. [71], self-reported level of walking frequency/time [75, 76], step counts and total walking activity (minutes per week). [74], and change in step counts/day from one visit to the next [72].
2. *Obesity*: Two studies [74, 77] assessed body mass index as an outcome. One study [77] further evaluated waist circumference.
3. *Cardiovascular Function*: Five papers [72–75, 77] measured systolic and diastolic blood pressures.
4. *Metabolic Syndrome*: One study [72] included in this review measured: Cholesterol and triglyceride levels, lipid profile (triglycerides, cholesterol, and HDL-C), and fasting blood glucose,

### Quality appraisal and risk of *bias* in included studies

Table 2 summarizes the risk of bias assessment for each of the included studies based on the PEDro scale. Further details are provided below:

**Table 2:** Quality Appraisal /Risks of Bias of Included Studies (PEDro Tool)

**Study**				**Sources/Potential sources of bias**	**Sources/Potential sources of bias**								
**Name**	**Eligibility** **Criteria**	**Random Allocation**	**Concealed Allocation**		**Baseline** **Similarity**	**Binding** **Of Sub-** **Jects**	**Blinding Of therapists**	**Blinding** **Of Accessors**	**Measures of Key Outcomes From 85% of the initially allocated**	**Intention** **To treat**	**Between** **Group**	**Point measure & variables**	**ROB** **Quality of study** **Grade Evidence**
**Furber et al. [2010]** [70]	**YES:**	**YES**	**YES**		NO	YES	NO	NO	No	NO	YES:	YES:	5/10/FAIR RISK MODERATE LEVEL 2
**McMurdo et al. [2010]** [71]	YES: .	YES:	NO		YES:	NO	NO	YES .	YES:	YES:	YES:	YES:	7/10/LOW RISK GOOD LEVEL 1
**Bjorgaas et al.** **[2008]** [72]	YES:	YES	NO		YES:	NO	NO	NO	NO	NO	YES:	YES:	4/10/FAIR RISK MODERATE LEVEL 2
**Kerr et al. [2018]** [73]	YES:	YES:	NO		NO	NO	NO	NO	NO	YES	YES:	YES:	4/10/FAIR RISK MODERATE LEVEL 2
**Kolt et al [2012]** [74]	YES:	YES	NO		YES:	NO	NO	YES:	NO	NO	YES	YES	5/10/LOW RISK MODERATE LEVEL 2
**Lee et al [2007]** [75]	YES:	YES;	YES		YES:	NO	NO	YES: .	YES:	YES:	YES	YES;	8/10/LOW RISK GOOD LEVEL 1
**Yamada et al. [2012]** [76]	YES: :	YES:	YES:		NO	NO	YES	NO	YES:	NO	YES:	YES:	6/10/LOW RISK GOOD LEVEL 1
**Yang & Petrini [2018]** [77]	YES:	YES:	NO		YES:	YES:	NO	NO	YES:	No	YES:	YES:	6/10/LOW RISK GOOD LEVEL 1
**OVERALL GRADE EVIDENCE**													5.625/10 LOW RISK MODERATE LEVEL 2

1. *Eligibility Criteria:* All eight studies [70–77] specified inclusion and exclusion criteria for recruiting and screening participants. Only older adults with modifiable risk factors for stroke were included, resulting in a low risk of bias in this section.
2. *Random Allocation:* All eight studies [70–77] outlined a randomization process for allocating eligible participants. This indicates a low risk of selection bias in this area.
3. *Concealment of Allocation:* Concealment of allocation was reported in three studies (37.50 %) [70, 75, 76]. However, it was not reported in five studies [71–74, 77], resulting in a 62.50 % prevalence of selection bias.
4. *Baseline Similarity:* Kerr et al.'s study [73] (12.5 % out of eight studies) included younger and married participants in the intervention group compared to the control group. Despite this difference, overall, there was a low risk of selection bias.
5. *Bias on Blinding:* Five studies (62.50 %) [70, 72, 73, 76, 77] reported assessor blinding. Three studies (37.50 %) [70, 75, 77] reported participant or personnel blinding, indicating performance bias.
6. *Intention-to-Treat Analysis:* Only three studies [71, 73, 75] reported intention-to-treat analysis, indicating a moderate risk of bias. Intention-to-treat analysis is essential for maintaining the integrity of RCTs.
7. *Between-Group Analysis and Point Measures/Variables:* All included studies conducted between-group analysis for control and intervention groups. Point estimates were used, and outcome variables were adequately measured.
8. *Bias of Outcome Measurement from < 85 % of Initial Participants (Incomplete Outcome Data* *): *All included studies reported follow-up of participants (Table 2).However, 14.6 % (226 out of 1475) of participants withdrew from studies, with withdrawal rates ranging from 5.7 % to 31.4 % within individual studies.Five studies [70, 71, 75–77] reported withdrawal rates above 15 %, indicating a high risk of incomplete outcome (attrition) bias.The studies included control groups with varying withdrawal rates above 15 %.In the comparative analysis of the clinical trial outcomes, the intervention cohort exhibited a marginally lower completion rate (*n* = 607) compared to the control cohort (*n* = 627), while the attrition rate was marginally elevated in the intervention arm (*n* = 106) relative to the control (*n* = 96). The statistical assessment of the proportional differences between the cohorts yielded a non-significant Z-score of -0.753, corresponding to a p-value of 0.226. This indicates that the observed variance has a 22.6% likelihood of occurrence under the null hypothesis, which posits no inherent difference between the groups. Consequently, the results do not provide sufficient evidence to reject the null hypothesis, suggesting that the observed differences in the attrition rates between the intervention and control cohorts could be attributed to random chance rather than a systematic effect attributable to the intervention.
9. *Evidence of Selective Reporting:*Only one [73] out of the eight studies in this review, was reported transparently by providing detailed information on outcomes related to physical activity, blood pressure, and physical functioning in both the intervention and control groups. The other seven studies [70–72, 74–77] had limitations in this area. Thus, one study [71], reported significant improvements in several outcomes (total physical activity sessions, walking minutes, walking sessions, cardiorespiratory fitness at 6 months, Psychosocial health at 6 weeks and 6 months) related to physical activity in the intervention group compared to the control group, but did not report negative or nonsignificant outcomes in detail. Another study [72] reported the intervention group did not show significant improvement in walking frequency compared to the control group, but did not provide detailed reporting on other outcomes (e.g., cardiovascular risk factors, glycemic control). One study [74] reported significant improvements in several outcomes (leisure Walking, overall physical activity and blood pressure) related to physical activity in the pedometer-based intervention group compared to the standard Green Prescription group, but did not report in detail the changes in body mass index across both groups or provide detailed reporting on other outcomes (e.g., quality of life, physical function, falls). Another study [76] reported significant improvements in several outcomes related to dependency in the intervention group compared to the control group, but did not provide detailed reporting on other outcomes (e.g., physical activity levels, psychosocial health). Another study [77] found Improvement in affect to exercise and physical activity behavior which was greater among participants in the self-selected intensity group vs. prescribed intensity but did not provide detailed reporting on other outcomes (e.g., cardiovascular risk factors, glycemic control). Overall, the lack of comprehensive reporting on all outcomes may introduce bias. The absence of such reporting could potentially indicate selective reporting bias.

### Outcomes reported in included studies

Eight studies [70–77] investigated the impact of pedometer-based walking interventions on physical activity, metabolic syndrome, and cardiovascular function. The aim was to understand the intervention’s influence on stroke risk factors.

### Effects of Intervention

The intervention’s effects are reported by comparing the intervention group to the control group, unless otherwise specified. Additional details are provided below:

- *Duration of Intervention*: The duration of the intervention across the studies ranges from 6 weeks [70] to 12 months [73, 74]. The mean trial duration was 7 months.
- *Intervention Progression*: Only one study [77] reported intervention progression: It varied from 30 - 50 minutes per session within the first 4 weeks. The duration remained unchanged for the remaining duration of the study.
- *Group vs. Individual Interventions*: Group intervention was used in three studies [73, 75, 76]. Five studies administered pedometer interventions individually [70–72, 74, 77].

The prescriptions that had positive outcomes were:

i. A 6-month self-monitoring of PA using a pedometer + step calendar + 15 minutes behavioural counselling & goalsetting sessions via telephone support given 1 wk. after pedometer INT. + two PA information brochures received by mail + booster phone calls at 12 weeks and 18 weeks [70].
ii. A 26-week pedometer-based walking programme + initial face-to-face advice on engaging in PA + 3 telephone counselling follow-up sessions (> 3 to 4mns) + Telephone counselling call 1 (Information provision & goalsetting - 15-30 mins) + call 2 (Assessing progress & further goalsetting - 10-15 mins) + call 3 providing further encouragement & discussions around relapse prevention - 10-15 mins) [74].
iii. Four individualized telephone counselling x 8 weeks & self-monitoring + Six-month pedometers walking intervention based on social cognitive theory and applied them in an Ecological framework, group education sessions, group walks, community advocacy & pedestrian community change projects [73].

#### Physical activity *outcomes*

Seven papers [70, 71, 73–77] included in this review (Table 1) reported on physical activity outcomes (Table 3). The majority [70, 71, 73, 74, 76] of the studies (5 out of 7, which is approximately 71.43%) found that pedometer-based walking programs significantly improved physical activity levels or outcomes in community-dwelling adults (with a *p*-value < 0.05). Notably, there was no significant decrease in value within or between groups in studies where no substantial improvement in physical activity outcomes occurred. Overall, the trend suggests that pedometer-based walking programs enhance physical activity outcomes in community-dwelling adults.

**Table 3:** Proof table for physical activity outcomes

**Study**	**Time point of measurement**	Outcome [Int. (Mean ± SD) vs Cont. (Mean ± SD); CI (…);* p*=…; d=…]	
Furber et al. [2010] (Australia) [70]	Immediately post-intervention	Total PA mins	Total PA mins. [pedometer grp (366.5 ± 270.8) vs control grp (270.9 ± 244.4); p = 0.027, d=0.31]
		Total PA sessions;	Total PA sessions [pedometer grp (9.0 ± 5.7) vs control grp (7.1 ± 5.6); *p* = 0.003, d=0.41]
		Walking mins	Walking mins [pedometer grp (249.9 ± 196.0) vs control grp (202.6 ± 189.5); *p* = 0.013 d=0.35],
		Walking sessions	Walking sessions [pedometer grp (7.2 ± 5.0) vs control grp (5.5 ± 4.0); (*p* = 0.002, d = 0.43)
Kolt et al (2012) New Zealand [74]	Baseline	Physical activity	Total walking activity min/wk: [Pedometer grp (81.5 (64.2 – 103.6) vs Standard grp (57.0 (44.3 – 73.3); *P*-= NR; D-value= NR; df= NR, *f*-value= NR
	3 months		Total walking activity min/wk: [Pedometer grp (106.1 (87.4 – 129.0) vs Standard grp (109.9 (87.9 – 137.6); P-= NR; D-value= NR; df= NR, *f*-value= NR
	12 months		Total walking activity min/wk: [Pedometer grp (143.0 (114.0 – 179.3) vs Standard grp (139.0 (112.0 – 172.5) *P*-= NR; D-value= NR; df= NR, *f*-value= NR Overall *p*-value= <.001
Kerr et al. [2018] USA [73]	Baseline	Physical activity (daily minutes) -Daily 3000-step increase for 12 weeks and maintain it	Int. (10.53 ± 13.58) vs [Cont. (6.76 ± 10.28) *p*= 0.02; D-value= NR; df= NR, *f*-value= NR
	3 months		[Int. (18.31 ± 22.58) vs [Cont. (6.54 ± 9.91) *P*-= NR; D-value= NR; df= NR, *f*-value= NR
	6 months		[Int. (15.60 ± 20.11) vs [Cont. (6.29 ± 8.56) *P*-= NR; D-value= NR; df= NR, *f*-value= NR
	9months		[Int. (12.87 ± 17.06) vs [Cont. (5.40 ± 7.83) *P*-= NR; D-value= NR; df= NR, *f*-value= NR
	12 months		[Int. (13.38 ± 16.87) vs [Cont. (5.96 ± 9.68) *P*-= NR; D-value= NR; df= NR, *f*-value= NR
McMurdo et al. [2010] Scotland [71]	Baseline	Accelerometry measurement (Minutes of Activity) 20% increase in step counts or mins in the first month & a further 20% at the end of the first and second months.	Minutes of Activity [Ped + BCI (180.2 ± 68.0) vs Cont. (159.6 ± 63.2; p = 0.04 BCI alone (160.9 ± 69.1) vs Cont. (159.6 ± 63.2; *p* = NR; df= NR, *f*-value= NR]
	Change recorded from BL to 3 months		Minutes of Activity [Ped + BCI (-1.31 ± 5.74) vs BCI alone (14.27 ± 6.42) vs Cont. (-5.86 ± 5.67; *p* = 0.05; df= NR, *f*-value= NR
Lee et al. [2007] Taiwan [75]	Baseline 6 months Post-Int	Self-reported level of walking frequency	Walking more: [Int. (48 ± 51.6) vs Cont. (8 ± 8.6) *P*-value= NR; df= NR, *f*-value= NR NO change: [Int. (43 ± 46.2) vs Cont. (71 ± 76.3) *P*-value= NR; df= NR, *f*-value= NR Walking less: [Int. (2 ± 2.2) vs Cont. (14 ±15.1) *p* value= NR; df= NR, *f*-value= NR Overall *P*-value= = *p*<0.0005)
Yang & Petrini et al. [2018] China [77]	Baseline	Physical activity -5,400 to 7,900 steps per day	Physical activity: [Self-selected Int grp (1.89 ± 0.27) VS Prescribed Int. Grp (2.03 ± 0.45) ) *p*-value= 0.337) ; df= NR, *f*-value= NR
	Post Intervention		[Self-selected Intensity grp (3.54 ± 0.64) VS Prescribed Intensity Grp (3.30 ± 0.61) *p*-value= 0.389) ; df= NR, *f*-value= 4.461
	3month follow-up		[Self-selected Intensity grp (3.61 ± 0.59) VS Prescribed Intensity Grp (3.33 ± 0.65) ) *p*-value= .026*) ; df= NR, *f*-value= 5.289)
Yamada et al.[2012] (Japan) [76]	Baseline 6 months Post-Int	Physical Activity (Steps)	[Int. (3726 ± 1607 vs control = NR (no mean value for the control was provided.

*BL* BASELINE, *CON* control, *grp* group, *NR* not reported, *NS* not significant, *df* degree of freedom, *b/w* between, *d* effect size

- Change in *Accelerometry Counts (Minutes Walking):*
  Five studies [71–73, 75, 76] conducted between-group and within-group analyses of step counts and associated physical activity. However, details of the within-group analysis were not fully reported in two studies [73, 76]. Additionally, two studies [70, 74] applied pedometer interventions, but did not use them to measure step counts as outcome. A high-quality study [71] revealed a significant increase in accelerometer counts within the Behavioral Change Intervention group compared to the control and pedometer PLUS Behavioral Change groups at the 3-month time point (Table 3). However, marginal reductions (*p* > 0.05) were observed at the 6th month. One moderate-quality study [73] reported a significant improvement in accelerometry step counts associated with moderate physical vigorous activity, which remained significantly higher than the control group at 3-, 6-, and 12-months follow-up time points. Additionally, only one study [76] reported within-group analysis of physical activity, while five other studies [70, 71, 73, 74, 76] provided within and/or between-group analyses. These findings emphasize the importance of considering both short-term and longer-term effects when evaluating interventions related to physical activity. Furthermore, including within-group analyses enhances our understanding of the dynamics within each study arm.
- Daily Step Counts: Two studies [76, 77] reported on the daily step counts (Table 1). In a high-quality trial [76], a significant increase (*p* < 0.05) in the daily step counts was observed in the intervention group compared to the control group (Table 3). Conversely, another high-quality study [77] found no significant difference (*p* > 0.05) in daily step counts between the self-selected and prescribed intensity groups.
- Self-Reported Level of Walking Frequency: The same two studies [75, 76] provided mixed results regarding daily step counts in the intervention group compared to the control group (Table 1). Specifically, the high-quality trial [76] reported a significant increase (*p* < 0.05) in the daily step counts of the intervention group (Table 3), while the other high-quality study [77] found no significant difference (*p* > 0.05) in daily step counts between self-selected and prescribed intensity groups.
- Physical Activity (Daily Minutes): A moderate-quality trial [73] demonstrated a significant increase in daily physical activity (measured in minutes) (*p* < 0.05) within the intervention group compared to the control group. This significant difference persisted throughout the study’s 12-month duration.
- Total Walking Activity (Minutes per Week: n a moderate-quality study [74], a significant (*p* < 0.05) increase in physical activity across all domains was observed at 3 months for both pedometer step-based Green Prescription and standard Green Prescription groups. This positive effect was maintained throughout the 12-month study (refer to Tables 2 and 3).
- Physical Activity Level: In a moderate-quality study [70], significant improvements (*p* < 0.05) were observed in the total physical activity time, total physical activity sessions, walking time, and walking sessions within the intervention group after 6 weeks (refer to Table 3). Remarkably, this effect remained significant even at the 6-month mark.

##### Comparison of intervention effects across sub grouped control groups:

Sub-grouping the studies revealed distinct patterns of results driven by each type of control group, as outlined below:

- *No-Contact Control Group:* Three studies [71, 75, 76] that measured physical activity (PA) in this subgroup demonstrated a remarkable 100 % improvement. Specifically, interventions (including pedometer-based programs and walking) led to enhancements in physical activity and self-efficacy. The impact varied based on the study and intervention types. Notably, the pedometer-based walking intervention was effective in increasing physical activity among older adults with hypertension [75], sedentary older women [71], and sedentary older adults [76].
- *Active Control Group:* Two studies [74, 77] within this subgroup, which measured PA indicators, found no significant change. This suggests that the overall health of the intervention and control groups did not differ significantly.
- *Social Control Group*: Among the three studies [70, 72, 73] in this subgroup, physical activity improved in only two (66.7%) studies [70, 73]. Consequently, the pedometer-based walking intervention effectively increased physical activity among cardiac patients [70] and older adults with no specific health conditions [73].

### Cardiovascular function (Blood Pressure)

Five studies [72–75, 77] provided data on mean resting blood pressure (Table 4). In a high-quality study [75], a significant decrease (*p* < 0.05) in systolic blood pressure was observed in both the intervention and control groups, with no difference in their diastolic systolic blood pressure levels (*p* > 0.05). Another high-quality study [77] found significant (*p* < 0.05) within-group differences in systolic and diastolic blood pressures in both intervention groups, with no significant (*p* > 0.05) between-group differences over time. Additionally, a moderate-quality study [74] identified significant differences in both intervention groups (*p* < 0.05), with no significant (*p* > 0.05) between-group differential change over time. Furthermore, one moderate-quality study [71] reported a decrease in diastolic blood pressure within the pedometer group, but no significant difference (*p* > 0.05) between the pedometer and no-pedometer groups. Another moderate-quality study [73] found a significant (*p* < 0.05) decrease in systolic and diastolic blood pressures at the 6^th^ month, which was no longer significant (*p* > 0.05) by the 12^th^ month. However, no data table was provided as evidence for this change.

**Table 4:** Proof table for Blood pressure

**Study**	**Time point of measurement**	Outcome [Int. (Mean ± SD) vs Cont. (Mean ± SD); CI (…); ***p***=, d=]	
Bjorgaas et al. [2008] **(** ***n*** **=69) **[72]	Baseline Immediately post-intervention	Blood pressure, Change Post-int.	Systolic BP: [Pedometer grp (-2.8 ± 17.3) vs Non-pedometer grp (-4.2 ± 25.5); *p*= NS; df= NS, *f*-value= NR Diastolic BP: [Pedometer grp (-2.9 ± 14.0) vs Non-pedometer grp (-7.4 ± 14.8); *p* = 0.048*; df= nS, *f*-value= NR
Kolt et al. [2012] **(** ***n*** **=330)** [74]	Baseline	Blood Pressure (mmHg)	Systolic BP [Pedometer grp (131.9 (127.1-136.7) vs Standard grp (133.4 (128.6 – 138.1) ) *P*-value= NR; D-value= NR; df= NR, *f*-value= NR Diastolic BP [Pedometer grp (77.4 ( 75.0 – 79.9) vs Standard grp (76.8 (74.3 – 79.4) *P*-value= NR; D-value= NR; df= NR, *f*-value= NR
	3 months follow up	3 months follow up	Systolic BP [Pedometer grp (134.8 (130.1-139.5) vs Standard grp (136.7 (132.0– 141.4) Diastolic BP [Pedometer grp (78.3 (75.8 – 80.8) vs Standard grp 78.8 (76.2 – 81.4)
	12 months follow-up	12 months follow-up	P-value of the systolic BP b/w the pedometer and Standard GRP over 12 months = *P*= <.001; D-value= NS, df= NS, *F*-valu=NS P-value of the diastolic BP b/w the Pedometer and Standard GRP over the 12 months = *P*= <.001; D-value= NS; df= NS,* f*-value= NS
Kerr et al. [2018] [73]	Baseline	Blood Pressure (mmHg) Baseline	Systolic BP [Int (132.06 ±19.24) vs CON grp (130.70 ± 19.07); *P*-value= 0.59; D-value= NR; df= NR, *f*-value= NR Diastolic BP [Int (69.24 ± 11.18) vs CON grp (67.00 ± 8.88); *P*-value= 0.81; D-value= NR; df= NR, *f*-value= NR
	6 months Post-Int	6 months Post-Int	Significant time x condition interaction at 6^th^ month Systolic BP (t value = − 2.68, *p* = .007) Diastolic BP (tvalue = − 2.35 *p* = .02)
Lee et al. [2007] **(** ***n*** **=202)** [75]	Baseline	Blood pressure (mmHg) 6 months follow up	Mean Resting Systolic BP: [Int. (136.2 ± 16.7) vs Cont. (143.6 ± 15.3); *p* value= 0.002**; Mean difference = −8.1 (−12.0 to −2.7) f-value= NR
	6 months Post-Int	6 months Follow up	Mean Resting Diastolic BP: [Int. (76.7 ± 12.3) vs Cont. (75.7 ± 11.6) *p* =< 0.19] Mean difference = −1.8 (−4.4 to 0.9) *f*-value= NR
Yang & PEtrini et al. [2018] **(** ***n*** **=60)** [77]	Baseline	Blood Pressure (mmHg),	
	Immediately Post Intervention	Post Intervention	Systolic: [Self-selected Intensity grp (120.3 ± 7.7) VS Prescribed Intensity Grp (119.77± 10.50) *P*-value= .308; *F*-value= 1.061; df= NS
	3month Post Intervention	3month follow-up	[Self-selected Intensity grp (119.4 ± 7.8) VS Prescribed Intensity Grp (120.1 ± 9.1) p-value b/w groups over the 3 months = 0.293; *F*-value= 1.127; df= NS
	Baseline		
	Immediately Post Intervention	Post Intervention	Diastolic: [Self-selected Intensity grp (77.9 ± 6.4) VS Prescribed Intensity Grp (75.85± 8.02) *P*-value= 0. 618; *F*-value= 0.252; df= NS
	3month Post Intervention	3 months Followup	[Self-selected Intensity grp (79.9 ± 6.6) VS Prescribed Intensity Grp (78.2 ± 7.4) p-value over the 3 months = 0.534; *F*-value= 0.391; df= NS

*CON* control, *grp* group, *NR* not reported, *NS* not significant, *df* degree of freedom, *b/w* between, d effect size

Intervention effects compared across sub-grouped control groups:

a *No-Contact Control Group*: One study [75], which measured blood pressure, reported improved systolic blood pressure among older adults with hypertension.
b *Active Control Group*: Two studies [74, 77] within this subgroup, which measured blood pressure – systolic and diastolic blood pressures - found no change. This suggests that the overall cardiovascular health of the intervention and control groups did not differ significantly.
c *Social Control Group*: Among the two studies [72, 73] that measured cardiovascular parameters, only one (50%) study [73] showed improvement in one cardiovascular function (systolic blood pressure). Thus, the pedometer-based walking intervention was effective in improving cardiovascular health in this context (Table 5).

## Metabolic outcomes

### Obesity

1. *Body Mass Index:*
  Two studies [74, 77] found no significant decrease (*p* > 0.05) in body Mass Index (Table 5).In a high-quality study [77], there was no significant decrease (p > 0.05) in the body Mass Index of the Prescribed Intensity group. However, no within-group or between-group evidence was provided. Another moderate-quality study [74] also found no significant change (p > 0.05) in body mass index, whether in the pedometer step-based Green Prescription or standard Green Prescription groups. However, no between-group comparison data were provided.
2. *Waist Circumference:*
  In a high-quality study [77], no significant difference (*p* > 0.050) in waist circumference was observed in either the self-selected or prescribed intensity groups (Table 5).
3. *Fasting Plasma Glucose:*
  A moderate-quality study [72] reported a significant decrease (*p* = 0.0033) in fasting plasma glucose within the pedometer group. However, no significant difference (*p* > 0.05) was found between the intervention and control groups (Table 6).
4. *Glycated Hemoglobin:*
  One moderate-quality study [72] found a significant (*p* < 0.05) decrease in glycated hemoglobin within the pedometer group. Interestingly, there was no difference (*p* > 0.05) between the pedometer and non-pedometer groups (Table 6).

**Table 5:** Proof table for BMI and waist circumference

**Study**	**Time point of measurement**	**Outcome [Int. (Mean ± SD) vs Cont (Mean ± SD); CI (…); ** ***p*** **=…; d=…]**	
Kolt et al. [2012] **(** ***n*** **=330)** [74]	baseline	BMI (Kg/m^2^)	
	3 months post-intervention	3 months	BMI: [Pedometer grp (27.2 (26.3 – 28.0) vs Standard grp (26.3 (25.6 – 27.0); (*p*-value= NR); *F*-value= NR; df= NR
	12 months post-intervention	12 months	BMI: [Pedometer grp (27.0 (26.2 – 27.8) vs Standard grp (26.4 (25.6 – 27.1) Overall *P* value b/w groups over the 12 months = 0.06
Yang & Petrini et al. [2018] **(** ***n*** **=60)** [77]	baseline	BMI (Kg/m^2^)	
	Immediately post-intervention	Post Intervention	
	3 months post-intervention	3month follow-up	[Self-selected Intensity grp (26.86 ± 2.47) VS Prescribed Intensity Grp(26.28± 2.22) *P*-value= NR; *F*-value= NR; df= NR
	baseline	Waist circumference (unit of measure not stated)	[Self-selected Intensity grp (26.86 ± 2.46) VS Prescribed Intensity Grp (25.73 ± 2.54) Overall p-value b/w groups over the 3 months = 0.227; *F*-value= NR; df= NR
	Immediately post-intervention	Post Intervention	[Self-selected Intensity grp (92.6 ± 9.3) VS Prescribed Intensity Grp(90.3 ± 7.6) *P*-value= NS; *F*-value= NR; df= NR
	3 months post-intervention	3 months Follow up	[Self-selected Intensity grp (91.3 + 8.9) VS Prescribed Intensity Grp (88.8 + 7.5) Overall *p* value b/w groups over the 3 months = 0.388

*CON=* control,* grp =* group,* NR = *not reported,* NS = *not significant,* df = *degree of freedom,* b/w = *between,* d = *effect size

**Table 6:** Proof table for Fasting plasma glucose

**Study**	**Time point of measurement**	Outcome [Int. (Mean ± SD) vs Cont (Mean ± SD); CI (…); *p*=…; d=…]	
Bjorgaas et al. [2008] **(** ***n*** **=69) **[72]	Immediately post intervention	Fasting plasma glucose (mmol/L)	Fasting blood glucose: [Pedometer grp (−0.31 ± 2.05) vs non-pedometer grp (−0.78 ± 2.34) (p-value= NR) (overall p-value b/w groups= 0.033)
		HbA1c	HBA1c: [Pedometer grp (−0.15 ± 0.76) vs non-pedometer grp −0.23 ± 1.35) (*p*-value= NR) (overall *p*-value b/w groups= 0.034)

*CON* control, *grp* group, *NR* not reported, *NS* not significant, *df* degree of freedom, *b/w* between, *d* effect size, *NB* Results are presented as Int. vs Cont group except where specified

### Lipid profile

1. *High-Density Lipoprotein (HDL): *In a moderate-quality study [72], a significant increase in HDL-C was observed in the pedometer group. Interestingly, there was no difference (*p* > 0.05) between the pedometer and non-pedometer groups (refer to Table 6).
2. *Triglycerides: *The same moderate-quality study [72] found a significant (*p* = 0.002) decrease in triglycerides within the pedometer group. However, there was no significant difference (*p* > 0.05) between the pedometer and non-pedometer groups (Table 7).
3. Cholesterol: In the same moderate-quality study [72], the cholesterol level was slightly higher in the pedometer group than in the non-pedometer group. However, it was not indicated whether the mean difference was significant or not (Table 7). Additionally, triglycerides were significantly elevated (*p* = 0.001) in the pedometer group compared to the non-pedometer group. Conversely, the HDL-C was significantly reduced (*p* = 0.001) in the pedometer group compared to the non-pedometer group.

**Table 7:** Proof table for cholesterol

**Study**	**Time points of measurement**	**Outcome [Int. (Mean ± SD) vs Cont (Mean ± SD); CI (…); ** ***p*** **=…; d=…]**	
Bjorgaas et al. [2008] (*n*=69) [72]	Immediately post intervention	Cholesterol (mmol/L)	Cholesterol: [Pedometer grp (0.17 ± 0.84) vs non-pedometer grp (0.15 ± 0.56) (p value= NR)
		HDL cholesterol (mmol/L),	HDL Cholesterol: [Pedometer grp (0.06 ± 0.03) vs non-pedometer grp (0.10 ± 0.15) (*p*-value= NR) (Overall p-value b/w groups= 0.001)
		Triglyceride (mmol/L)	Triglyceride {Pedometer grp (1.17 ± 0.32) vs non-pedometer grp (-0.23 ± 0.64) (*p*-value= NR) (Overall *P* value b/w groups= 0.002)

*CON* control, *grp* group, *NR* not reported, *NS* not significant, *df* degree of freedom, *b/w* between, *d* effect size

Results are presented as Int. vs Cont group except where specified

#### Intervention effects compared to sub-grouped control groups

##### Social control group

Sub-grouping the included studies according to the type of control group revealed that only the social control group was used by the two studies [72, 73] that measured metabolic outcomes. Only one (50%) study [73] showed improvement in various metabolic parameters, including weight, body mass index, waist-to-hip ratio, fasting plasma glucose, glycated hemoglobin, total cholesterol, HDL-cholesterol, triglycerides, VO_2_peak (L/min), and VO_2_peak (mL/[kg min]). Notably, the pedometer-based walking intervention was effective in improving metabolic parameters in older adults with no specific health conditions [73].

### *Meta*-analyses – effects of interventions

**Fig. 2:**
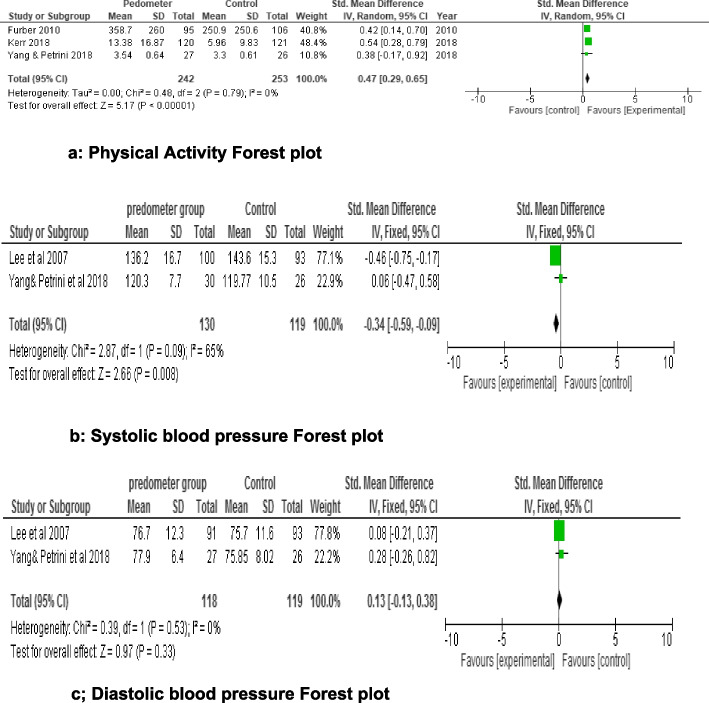
Physical Activity Forest plot In the primary meta-analysis, **a** significant effect of the pedometer-based walking intervention on physical activity levels was observed (SMD = 0.47, 95 %. Confidence Interval [CI]: 0.29 to 0.65, Z = 5.17; *p* < 0.00001; 4 studies; 532 participants). There was low statistical heterogeneity (I^2^ = 0%, χ^2^ = 0.48, df = 2, *p* = 0.79). **b** Systolic blood pressure Forest plot **c**, Diastolic blood pressure Forest plot

The meta-analyses included four studies [70, 73, 75, 77], while four studies [71, 72, 74, 76] were excluded. The exclusions occurred because means and standard deviations were not reported for fasting blood sugar and diastolic blood pressure in one study [71], and for recording changes in fasting blood sugar, body mass index, physical activity, systolic and diastolic blood pressure without stating them in means and standard deviations [80]. Additionally, two high-quality studies [71, 75] failed to provide post-intervention means and standard deviations for physical activity variables assessed [71], as well as walking frequency, diastolic and systolic blood pressures [75]. Furthermore, one study [76] did not provide baseline and post-intervention mean of physical activity (steps) for the control group. Consequently, this review conducted three meta-analyses (Fig 2a–c) for studies that evaluated physical activity level, systolic and diastolic blood pressures, with more than two studies meeting the criteria for meta-analyses.

#### Intervention *effects* and subgroup analysis

##### Subgroup analysis

Subgroup analysis of the included studies according to the type of control group for each study outcome was not feasible, as the studies that met the criteria for inclusion into the meta-analysis were not up to two per sub-group (Fig. 2).

### Physical activity level

Two moderate-quality studies [70, 73] out of three [70, 73, 77] reported a significant increase in the physical Activity Level post-intervention. However, one high-quality study [77] reported no change. The included studies used pedometers [77], accelerometers [73], and The Active Australia Questionnaire for self-reported physical activity [70] (Table 1) to measure physical activity. Notably, physical exercise training prescriptions that improved outcomes were:

i. A 6-month self-monitoring of PA using a pedometer + step calendar + 15 mins behavioural counselling & goalsetting sessions via telephone support given 1 wk. after pedometer INT. + two PA information brochures received by mail + booster phone calls at 12 wks. & 18 wks [70].
ii. A 26-week pedometer-based walking programme + initial face-to-face advice on engaging in PA + 3 telephone counselling follow-up sessions (> 3 to 4mns) + Telephone counselling call 1 (Information provision & goalsetting - 15-30 mins) + call 2 (Assessing progress & further goalsetting - 10-15 mins) + call 3 providing further encouragement & discussions around relapse prevention - 10-15 mins) [73].
iii. Aerobic walking or jogging at Self-selected intensity or prescribed moderate intensity, 3x/week for 6 months duration with 24 hrs intervals between sessions; monitored by the RPE scale, 50 min/session; volume was set as 5,400 - 7,900 steps/dy.; progression was an initial 30 min/session @ the 1st wk & a 5-min increase in session time/wk over the first 4 weeks until up to 50 min/session [77].

In the primary meta-analysis (Fig. 2a), a significant effect of the pedometer-based walking intervention on physical activity levels was observed (SMD = 0.47, 95 % Confidence Interval [CI]: 0.29 to 0.65, Z = 5.17; *p* < 0.00001; 4 studies; 532 participants). There was low statistical heterogeneity (I^2^ = 0%, χ^2^ = 0.48, df = 2, *p* = 0.79).

#### Systolic blood pressure

Two studies [75, 77] showed divergent results regarding systolic blood pressure metrics. Specifically: one high-quality study [75] demonstrated a significant decrease. Conversely, a moderate-quality study [73] reported a significant reduction at six months, but not at 12 months. However, the study [73] did not provide data on adherence rates after 6 months and did not disclose participants’ historical usage of anti-hypertensive or antidepressant medications. Despite the lack of comprehensive data on adherence rates and medication usage, certain pedometer-based walking intervention programs have demonstrated antihypertensive effects, as described below:

I. A four individual telephone counselling x 8 weeks & self-monitoring + Six-month pedometers walking intervention based on social cognitive theory and applied them in an Ecological framework, group education sessions, group walks, community advocacy & pedestrian community change projects [75].

#### Systolic blood pressure

A primary meta-analysis (Fig. 2b) found a significant effect of pedometer-based walking intervention on systolic blood pressure with a small standardized mean difference (SMD = -0.34, 95 % CI: -0.59, -0.09; Z = 2.66, *p* = < 0.008; 2 studies; 249 participants), and moderate statistical heterogeneity (I^2^ = 65 %, χ^2^ = 2.87, df = 1, *p* = 0.09). Measuring tools used in the included studies were: sphygmomanometer (Table 1).

#### Diastolic blood pressure

Two high-quality studies [75, 77] included in the meta-analyses found significant within-group decreases in diastolic blood pressure for both intervention and control groups. The pedometer-based walking prescriptions that reduced diastolic blood pressure in the two studies were:

iii. A-four individual telephone counselling x 8 weeks & self-monitoring + Six-month pedometers walking intervention based on social cognitive theory and applied in an Ecological framework, group education sessions, group walks, community advocacy & pedestrian community change projects [75].
iv. A 6-month aerobic walking or jogging @ Self-selected intensity or prescribed moderate-intensity, 3x/week for 6 months duration with 24 hrs intervals between sessions; monitored by the RPE scale, 50 min/session; volume was set as 5,400 - 7,900 steps/dy.; progression was an initial 30 min/session @ the 1st wk & a 5-min increase in session time/wk over the first 4 wks until up to 50 min/session [77].

A primary meta-analysis (Fig. 2c) found no significant effect of pedometer-based walking intervention on diastolic blood pressure with a small standardized mean difference (SMD = 0.13, 95 % CI: -0.13, 0.38; Z = 0.97, *p* = 0.33; 2 studies; 237 participants), and low statistical heterogeneity (I^2^ = 0 %, X^2^= 0.39, df = 1, *p* = 0.53 (Fig. 2c - forest plot). Measuring tools used in the included studies were: sphygmomanometer. (Table 1).

### Grade of evidence for the review

The review found that 75% (or six) of the eight studies included in the review were graded as level 1 (good grade evidence) using the PEDro assessment method. Additionally, two studies [72, 73] were rated as level 2 (poor grade evidence). The overall grade point evidence (Table 2) for the review is 6.86 out of 10, which corresponds to level 1 (good grade evidence) for estimating the effects of pedometer-based walking on the study outcomes.

### Theoretical synthesis

#### Effectiveness of behavior theories in pedometer-based physical activity interventions for community-dwelling older adults

**Fig. 3:**
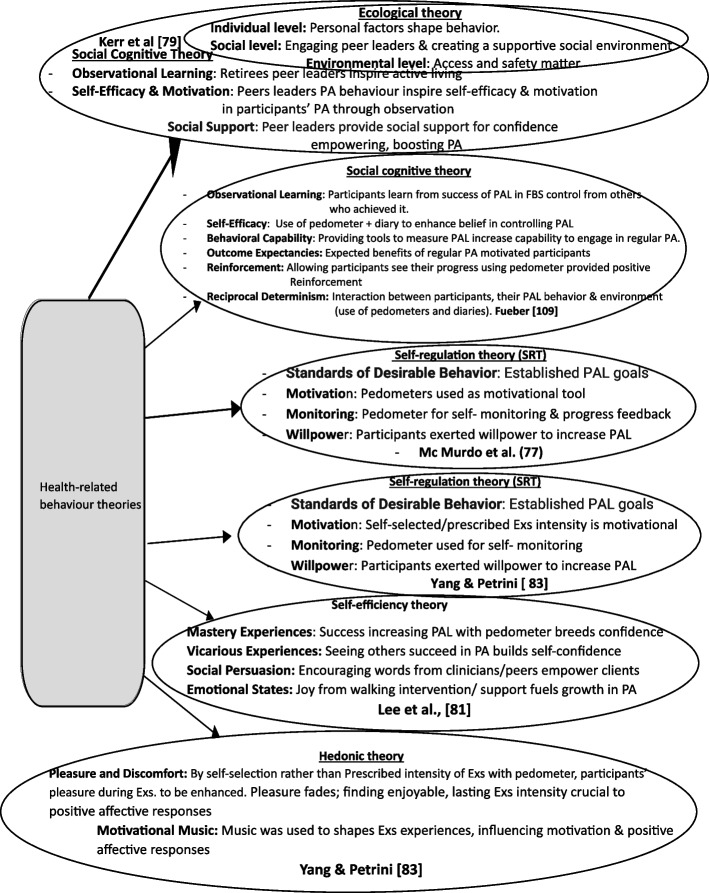
Mapping of the health-related behaviour theories for pedometer-based physical activity; PA = Physical activity; Exs = Exercises; PAL = Physical activity level

Five studies [70, 71, 73, 75, 76] utilized behavior change theories to design or justify behavior change interventions related to pedometer-based physical activity. Although these studies aimed to enhance adherence behaviors in pedometer-based physical activity, their specific goals varied. Consequently, the narrative data was combined to align with evaluation goals, providing a framework for presenting findings. First, a map of research on pedometer-based physical activity interventions to enhance physical activity behavior in older adults using behavior modification theories or models was created (see Fig. 3). Subsequent theoretical synthesis focused on studies that utilized behavior change theories or models to design and/or evaluate the efficacy of pedometer-based walking programs in enhancing physical activity behavior among community-dwelling older adults. The following points summarize the findings:

i. *Disease Staging in Community-Dwelling Older Adults and Theory-Based Interventions:*
  Among the five studies using theory-based interventions, only one study [70] found poor cardiac health status in 59 (26.57%) of the 202 older adults with cardiac diseases. The lack of disease diversity measurement (i.e., disease stage) may impact behavior change models, specifically influencing incentives or demotivation to act.
ii. *The Theoretical Basis of Pedometer-Based Physical Activity Behavior Interventions*
  The pedometer-based physical activity behavior interventions in all five studies [70, 71, 73, 75, 77] were based on different health-related behavior change theories or models with details provided below:
  a *Dual Theories or Models:*
    In one study [73], the intervention was informed by two theories or models: social cognitive theory and ecological theory. Another study [77] explained the impact of the intervention on physical activity behavior using two theories: hedonic theory and the dual-mode model.
  b *Single Theory* *or Model:*
    Three studies [70, 71, 75] utilized a single theory or model to inform the pedometer-based physical activity behavior modification treatments. Specifically, one study [75] was based on the self-efficacy theory, while another [71] was based on the self-regulation theory.
  c *Social Cognitive Theory Application:*
    Two studies [70, 73] provided comprehensive information on how they differently utilized the social cognitive theory in developing the pedometer-based physical activity behavior intervention. In one study [73], social cognitive theory techniques were applied within an ecological framework. Interventions occurred at the individual (goalsetting), interpersonal (group walks), and community levels (with a focus on pedestrian advocacy for improved walkability). Peer leaders played a crucial role in delivering the physical activity intervention program. The other study [70] centered around the social cognitive theory construct of self-efficacy. The intervention design emphasized goalsetting and self-monitoring to enhance participants’ perceptions of the positive health outcomes of physical activity, aiming to increase their belief in its benefits.
  d *Self-Efficacy Theory Application:*
    In one study [75], the self-efficacy theory constructs were applied in designing and measuring the outcome of the physical activity intervention.
  e *Dual Theory Application:*
    One study [77] applied two theories to explain the impact of the pedometer on physical activity behavior.
iii. *Theory and the Physical Activity Behavior Intervention Design:*
  Among the studies, only three [70, 71, 75] applied a single behavior change theory or model to design pedometer-based physical activity behavior interventions. However, other studies utilized more than one theory. Notably, one study [70] did not include an important component that would require increased participants’ belief in the health benefits of physical activity as a motivator to enhance self-efficacy and goalsetting for the physical activity intervention. Additionally: One study [73] used two health-related theories to design a pedometer-based intervention. Another study [77] used one theory and a model to explain its impact on physical activity behavior and affective responses to walking.
iv. *Application of Health-Related Theory to Results Assessment:*
  In one study [73], which used social cognitive theory within an ecological framework, the analysis focused on social determinants of health and their interaction with physical activity. This included exploring the influence of others in the group and community. Another study [70] assessed participants’ perception of pedometers as motivators for physical activity goals, based on social cognitive theory. However, this study did not explore participants’ knowledge of health benefits. The self-efficacy theory was used in one study [75] to assess participants’ confidence to exercise in the face of barriers (such as bad weather and tiredness) and self-efficacy expectations when using a pedometer as a motivator for exercise. Additionally, one study [70] utilized the hedonic theory to analyze the impact of pedometer-based programs on physical activity behavior and the dual-mode model to explore affective responses to exercise intensity.
v. *Targeted Subpopulations or Populations:*
  Five research studies examined community-dwelling older adults, focusing on specific populations without any specific health conditions. These populations included individuals with cardiac diseases [70], hypertension [75], non-fallers [73], sedentary lifestyles [71], and inactive retirees [77]. The studies included in the review targeted more than one demographic of older adults, ranging from those without specific conditions [73] to either inactive older adult retirees [77] or sedentary older women [71] or older adults with cardiac diseases [70] and older adults with hypertension [75]. Notably, the target demographics were not divided, and alternative therapies were not offered to participants in specific groups.
vi. *Categories of Behavior Targeted by Pedometer-Based Physical Activity Interventions:*
  In five studies [70, 71, 73, 75, 77], physical activity level was the most frequently targeted behavior for modification. Efforts to alter physical activity behavior (including total physical activity in minutes, total physical activity sessions, walking time in minutes, and walking sessions) were based on self-monitoring of physical activity level using pedometers [70] and accelerometry counts [73]. Motivation for increased physical activity was boosted through individual counseling, self-monitoring using pedometers, group education and walks, community advocacy, and pedestrian community change projects. Included studies attempted to increase walking among participants through various methods, including:
  Using a pedometer and walking log, and boosting through community-based walking, face-to-face interaction, and phone calls [75].
  Conducting brief education sessions focusing on beliefs and motivation for walking, bolstered by a self-regulation approach based on goalsetting, action and coping planning, self-monitoring, and feedback inputs for the desired change in physical activity behavior [71].
  Prescribing and self-selecting intensities and monitoring using the Borg Rating of Perceived Exertion scale [77].
vii. *Health Communication Channels, Activities, and Settings Used in the Studies:* Although health communication was not an inclusion criterion, this review found that all five studies [70, 71, 73, 75, 77] described some health communication channels or interaction activities with participants. These included: telephone calls and emails (recorded in the study log) [70], phone calls and text messages [77], face-to-face interaction/interviews and telephone calls to provide motivation, encouragement, and troubleshooting [71], telephone calls to identify barriers and support safe goalsetting [73]. The studies were conducted in various settings, such as retirement residential homes [73], home settings [70, 71], community playgrounds [77], and community activity centers [75]. The most common interaction activities were face-to-face interaction/interviews [73, 75] and telephone calls [70, 71, 73, 75, 77].
viii. *Applicability of the Theory or Model in the Intervention:*
  Three out of the five studies [70, 73, 75], based on health-related behavioral theories, assessed the applicability of the theory or model with the following details:
  - *Self-Efficacy Theory Application: *In one study [75], self-efficacy theory was used to evaluate physical activity behavior change.Another study [70] assessed the impact of social cognitive theory on self-efficacy, outcome expectancies, setting physical activity goals, and the use of behavioral and cognitive self-management strategies in the intervention group at the time points where physical activity goals had been set.
  - *Self-Regulation Theory Application:*Two studies [71, 77] based on self-regulation theory (which emphasizes the role of goalsetting, planning, and self-monitoring behavior change), failed to apply its principles in evaluating the findings.Specifically, one study [71] required participants to meet a 20 % increase in pedometer step counts or minutes walked but did not evaluate how this contributed to physical activity behavior change. The authors did not detail how the theoretical constructs applied to their interventions were used in developing any of the evaluation tools or how meeting the set targets by the participants at each time point contributed to the physical activity behavior change.
  - *Hedonic Theory Application: *One study [77] used the hedonic theory as a framework to explain how individuals’ affect toward exercise impacts exercise behavior. However, this study did not develop or evaluate the interventions based on the theory.
ix. *Achievement of Health Behavior Change Objectives in Pedometer-Based Physical Activity Interventions*
  Five high-quality studies [70, 71, 73, 75, 77] were reviewed, with two studies [75, 77] or 40% of them graded as high quality (≥ 75% overall validity). Notably: only one high-quality study [75] demonstrated significant behavior changes, primarily related to physical activity. Three studies [70, 71, 73] targeted participants in residential/home settings. Four studies [70, 71, 73, 75] showed significant modification in physical activity behavior, while one study [77] showed no significant change. This review found that three individual-level behavior theories—namely, self-efficacy [75], self-efficiency [71], and social cognitive [70] theories—positively impact physical activity behavior outcomes. Additionally, the combination of an individual-level theory (Social Cognitive Theory) with a community-level behavior theory (applied within an Ecological Framework) improved physical activity behavior in one study [73]. However, the combination of hedonistic theory and the dual-mode model was not associated with a significant change in physical activity behavior and was not used for intervention design in the only study [77] that applied them.
x. *Evidence for Effective Interventions and Associated Theories/Models of Behavior Change to Improve Physical Activity Behaviors, Prevent, or Control Stroke Induction and/or Progression*:
  One high-quality study [77] aimed to increase physical activity behaviors and improve affect toward exercise, body mass index, waist circumference, and blood pressure. However, it did not specifically focus on stroke prevention or control. Therefore, it was challenging to determine whether the pedometer-based physical activity behavior intervention would be effective not only in achieving the primary goal of improving physical activity behavior but also in reducing risk factors for stroke, such as body composition indices (waist circumference, body mass index, lipid profile, blood pressure, and metabolic diseases (e.g., hyperglycemia). One study [75] found a positive change in systolic blood pressure as a primary outcome, and similarly, another study [73] measured blood pressure (both systolic and diastolic blood pressure) as a secondary outcome. Among all four studies [70, 71, 73, 75] whose pedometer-based interventions were underpinned by behavior change theories, no comparative evidence was provided to evaluate whether utilizing the theory made the pedometer-based intervention effective or not. However, by mapping and matching the studies according to the underpinning theory, a useful comparison between those with an effective pedometer-based intervention (i.e., successful outcomes) and those without was enabled.
  One study [71] exclusively employed pedometer-based walking programs, whereas seven other studies[70, 72–77] integrated these programs with face-to-face advice, goal-setting for walking and dance activities, behavioral change interventions, 15-minute reinforcement telephone calls, behavioral counseling, and goalsetting sessions, as well as group education sessions, individual counseling, pedestrian community change projects, and community advocacy.

## Discussion

Eight studies [70–77] evaluated the effectiveness of pedometer-based walking programs in improving modifiable risk factors for stroke among the community-dwelling older adult population. This review included studies with a low to moderate risk of bias, mostly of high or moderate methodological quality, and was conducted in high-income (developed) countries, with none from developing countries. The absence of studies from developing countries in this review could have implications, considering that stroke burden has shifted from developed to developing countries. Developing countries now account for 75 % of all stroke deaths and 81 % of total disability-adjusted life years lost due to stroke [103]. The epidemiological shift in stroke burden from developed to developing countries is driven by population aging, population growth, and changing disease patterns due to risk factor modifications and differences in socioeconomic status and healthcare [104, 105]. Despite a 30-45 % decline in stroke rates in Europe from 1975 to 2005 [106], stroke still accounts for 10-12 % of all deaths in developed countries [107]. Therefore, the results of this review hold significance for both developed and developing countries.

### Physical activity

This review focuses on improving physical activity behavior and addressing diseases using pedometer-based strategies. While Yang & Petrini [77] had two exercise groups (Self-Intensity and Prescribed Intensity) and reported no significant difference in the daily steps program, similar to Bjørgaas *et al.,* [72], six other studies [70, 71, 73–76] demonstrated significant improvement in physical activity variables. These findings highlight trends for clinicians exploring pedometer-based walking programs among community-dwelling older adults.

i. *Differences in Physical Activity Improvement Based on Control Groups:*
  The observed differences between two sets of studies—one with significant improvement in physical activity behaviors [70, 71, 73–76] and the other without [72, 77]—can be attributed to the type of control groups employed. Notably, all studies that employed no-contact control groups [71, 75, 76] reported significant improvement in the participants’ physical activity levels. Additionally, most studies that used social control groups [70, 73] and one (half) of the two studies that used active control groups [74] also showed significant enhancement in the physical activity levels of participants. The only study [74] with an active control group that showed a significant change in physical activity behavior received same green prescription intervention as the experimental group except that they were provided counselling focused on accumulating physical activity around time-related goals rather than step-related goals and without a pedometer to monitor number of steps. The only study [72] that incorporated social control but did not demonstrate a significant change in physical activity had control group who were not provided with pedometers; instead, they received guidance to accumulate physical activity based on time-related goals. The advice aimed to increase the average daily time spent walking between visits. Major physical activities were meticulously recorded in a logbook, and individual strategies to enhance walking were established and discussed with the study nurse. Evidently, the study with active control lacked components such as goal monitoring, motivation, self-regulation, and advice on establishing strategies to increase physical activity within the environmental context. In contrast, the study with social control included these components, suggesting their potential role in enhancing physical activity behavior change, even in the absence of pedometers. Similarly, the absence of significant differences in physical activity levels between the experimental and active control groups does not necessarily indicate a lack of effect. Instead, it highlights those alternative therapies positively influenced the physical activity levels of the control group, thereby mitigating the apparent impact of the pedometer-based intervention when compared to the active control group. The same principle applies to the social control group. Therefore, when interpreting RCTs in the literature, these factors should be carefully considered.
ii. *Evidence Supporting Pedometer-Based Programs*
  The findings of this review align with a meta-analysis [108] that concluded pedometer-based interventions and found an increase in physical activity levels across different sexes and age groups. However, the effects vary for older adults, adults, and children, and an intervention strategy may not be universally appropriate for all age groups. Similarly, step-count monitoring interventions [109] lead to improved step counts over 6 months and 1 year, providing an additional 1050 and 464 steps per day, respectively. Two pedometer-based walking trials [41, 110] demonstrated long-term increases in physical activity over four years, along with reduced cardiovascular events and fractures [102]. Additionally, a study by Croteau et al. [111], which examined step count interventions and their maintenance effects during a 12-week follow-up, found that participants increased their daily step counts during the maintenance period. Overall, the majority of evidence from the included papers in this review supports the positive impact of the pedometer-based walking programs in improving physical activity behavior, with no adverse events documented, indicating their safety and utility as lifestyle interventions. The six other studies [70, 71, 73–76] that demonstrated significant improvements in physical activity variables are particularly relevant for clinicians exploring pedometer-based walking programs among community-dwelling older adults.

### Theoretical frameworks for pedometer-based walking interventions: insights and implications

In the context of pedometer-based walking interventions, this review underscores the significance of three theoretical frameworks: *Self-Efficacy Theory* ***,*** *Social Cognitive Theory, and the Ecological Framework* **.** Among these, the application of Social Cognitive Theory within an ecological context has yielded substantial changes in physical activity levels. This outcome is likely attributed to the theory’s contextualized approach. The findings of this review suggest that pedometer-based walking interventions should play a pivotal role in health promotion and stroke prevention among community-dwelling older adults. Understanding the mechanisms underlying behavior change is essential for designing effective strategies. The fact that the use of Social Cognitive Theory within an Ecological Framework resulted in the greatest change in physical activity further highlights the importance of taking social determinants relevant to diverse populations into account, especially in Africa and other regions where pedometer-based interventions have not yet been thoroughly investigated. By considering the dynamic interconnections between different environmental systems (such as family, community, peers, and culture), this paradigm expands on Social Cognitive Theory. It is plausible that integrating theory, considering social determinants, and contextualizing interventions contribute to successful physical activity promotion.

i. *Interpersonal Health Behavior Theories and Pedometer-Based Interventions*
  Though this review emphasizes the significance of three theoretical frameworks—the Ecological Framework, Social Cognitive Theory, and Self-Efficacy Theory—within the context of pedometer-based walking therapies, among these, the application of Social Cognitive Theory within an ecological context holds promise for diverse populations [112, 113] in Africa, emphasizing the need for culturally sensitive approaches. Effective pedometer-based interventions in African cultural communities require peer-led approaches and cultural sensitivity. Other socio ecological-based models that can be applied to investigate the effects of pedometer-based walking include Bronfenbrenner’s Social-Ecological Model [112], the Health Belief Model [114], and the Trans-Theoretical Model [115]. The Bronfenbrenner model [112] emphasizes multilevel influences on behavior highlighting the interplay of various human development systems, including microsystems (individual factors such as family support), mesosystems (interpersonal factors like collaboration with schools), exosystems (like organizational or environmental factors), macrosystems (community related factors like cultural norms), and chronosystems (policy factors like life transitions). While not purely socioecological, Health Belief Model, a socioecological approach, considers individual perceptions, cues to action, and self-efficacy to inform pedometer-based interventions, addressing perceived benefits, barriers, and self-efficacy related to walking. Trans-Theoretical Model [115] involves stages of behavior change, including precontemplation, contemplation, preparation, action, and maintenance, and can be customized based on an individual's readiness to adopt walking as a regular activity.
  However, four studies [70, 71, 73, 75] in this review, which examined pedometer-based physical activity behavior change strategies based on Social Cognitive Theory, found significant changes in physical activity behavior among community-dwelling older adults. Three studies [73, 74, 76] analyzed components of self-regulatory theory for interventions. Interestingly, the only studies that omitted environmental components [72, 77] showed no significant change in physical activity behavior. This suggests that the ecological barriers to implementing the pedometer-based intervention were not identified and addressed. While three studies [72, 74, 76] applied self-regulation theory components, it was not explicitly mentioned that the theory informed the design of the pedometer-based intervention strategy. In contrast, one study solely used hedonic theory and the dual-mode model to explain but not design the pedometer-based intervention, resulting in no change in physical activity outcomes. Social Cognitive Theory, as applied by Furber et al. [70] and Kerr et al. [73], suggests that individuals have a sense of agency [71, 116, 117], and control over their lives. Counseling and training sessions on physical activity can enhance this sense of agency. In this regard, the self-regulatory theory applied by McMurdo et al. [71, 116, 117] and the self-efficacy theory applied by Lee et al., [75] fit into the broader context of social cognitive theory. Both self-regulation and self-efficacy serve as pathways to experiencing a greater sense of agency or agentic perspective [113, 118].
ii. *Enhancing Physical Activity Among Older Adults: Insights from Interpersonal Health Behavior Theories*
  Several studies [71, 73, 75] demonstrate that interventions grounded in interpersonal health behavior theories yield successful outcomes in enhancing physical activity behavior among community-dwelling older adults. Notably, these theories include self-regulatory theory, self-efficacy theory, and a combination of both within an ecological framework. Interestingly, this finding contradicts an earlier perspective that multiple-theory interventions might be less effective than single-theory interventions [119, 120], however, the reasons behind this assumption remain unelucidated. However, it is essential to recognize that unsuccessful studies [72, 77] did not adequately account for environmental factors or the impact of personal variables on physical activity behavior. In contrast, the same studies revealed that pedometer-based interventions significantly improved physical activity behavior and reduced risk factors related to stroke. These interventions, spanning durations from 6 weeks to 12 months, led to improved indicators for stroke prevention. Interestingly, the unsuccessful studies [72, 77] shared a similar duration (that is 6 months) with the successful ones indicating that the duration of the trials did not inform the differences in results. Consequently, these findings underscore the critical importance of designing pedometer-based interventions based on interpersonal health behavior theories. Neglecting this aspect may diminish the overall effectiveness of such interventions.

### Pedometer-based walking and blood pressure control in older adults

This review also emphasizes the importance of pedometer-based walking in controlling hypertension and preventing stroke among community-dwelling older adults. Epidemiological data suggest that older adults are more likely to experience white-coat hypertension**,** isolated systolic hypertension**,** and pseudo-hypertension [121]. The findings from this meta-analysis indicate that pedometer-based walking programs can lead to a statistically significant reduction in systolic blood pressure by 0.34 mmHg. However, the reduction in diastolic blood pressure (0.13 mmHg; *p* = 0.33) did not reach statistical significance. While the effect size may appear modest at an individual health level, it is crucial to consider its broader impact on population health. Even small reductions in systolic blood pressure could have meaningful implications for older adults with hypertension. Therefore, recommending pedometer-based walking as a clinical tool for controlling and preventing high blood pressure warrants further consideration. This has significant implications for older adults with high blood pressure, especially considering that as adults get older, systolic blood pressure tends to increase while diastolic blood pressure tends to decrease, leading to isolated systolic hypertension. Isolated systolic hypertension is defined by an average systolic blood pressure ≥ 140 mmHg [121] and diastolic blood pressure < 90 mmHg [122], and it becomes more relevant for older adults aged > 60 years. Therefore, the significant reduction in systolic blood pressure observed in this review following pedometer-based physical activity intervention hints at its potential anti-hypertensive effect for the prevention and control of stroke. It may also alter the prognosis of stroke recurrence**.** The pedometer-based physical activity program may also help relieve White Coat Hypertension or Isolated Office Hypertension. These conditions are defined by persistently elevated average office blood pressure > 140/90 mmHg in addition to an average awake ambulatory blood pressure < 135/85 mm Hg. White Coat Hypertension or Isolated Office Hypertension is found in 15%–20% of individuals diagnosed with stage 1 hypertension [123]. However, the responses of individuals with masked hypertension or isolated ambulatory hypertension [124], pseudo hypertension [121], and orthostatic or postural hypotension require further investigation [125]. The pedometer-based walking program may not be effective in controlling isolated diastolic hypertension (defined by systolic blood pressure < 140 mmHg and diastolic blood pressure ≥ 90 mmHg) [126], which is a common condition in young adults under 50 [122]. However, it can be effective for the epidemiological spectrum of hypertension in older community-dwelling adults over 65 years. This view is supported by a previous finding [127] that demonstrated a significant decrease in systolic blood pressure (3.8 mmHg; *p* = .001) and diastolic blood pressure (0.3 mmHg; *p* = .001), independent of age, body mass index, or intervention duration. Another study [101] also revealed a significant reduction in systolic blood pressure levels at the end of a 59-week pedometer-based walking program. These findings suggest that pedometer-based walking could be an effective strategy for preventing stroke through its anti-hypertensive or ameliorative effects**.**

### Metabolic syndrome and pedometer-based walking: unraveling the complexities:

i. *Body mass index and pedometer-based interventions:*
  This review found no significant change in body mass index for the included studies. Yang & Petrini [77] reported no significant decrease between the two exercise groups, and likewise, Kolt et al. [74] observed that body mass index did not increase in both studies at 3 months and beyond. However, the findings of this review differ from other studies in the literature. A previous study [128] demonstrated that a 3-month pre-posttest walking program significantly improved body mass index, with an interaction effect between group and time differences. The study recommended pedometer-assisted self-monitored walking for older adults to cultivate long-term exercise habits and supervised walking to maximize effective exercise intensity. A meta-analysis [129] indicated that pedometer-based walking programs can cause modest weight loss, with longer programs leading to more substantial weight loss. However, this review involved a small sample of 323 participants across the two included studies, with one study’s [74] attrition rate exceeding 15 %. These flaws weaken our confidence in the estimate of effect; hence, no valid scientific opinion can be drawn from the review regarding body mass index. Further high-quality RCTs would be necessary to address this gap.
ii. *Exploring waist circumference and pedometer-based interventions:*
  This review included only one study [77], which found no significant decrease in waist circumference in the Prescribed Intensity Exercise group. However, a previous study [130] revealed an interaction effect between the activity group and time concerning the waist circumference variable (*p* = 0.048). Participants who achieved 20 minutes of physical activity daily showed a decrease in waist circumference (98.7 cm to 96.2 cm, *p* = 0.003), unlike those who did not achieve this landmark (100.5 cm to 100.0 cm, *p* = 0.38). While this review lacks sufficient data to form a valid scientific opinion, the trends suggest that walking with its health benefits, should be encouraged for those struggling with weight loss [128], especially among community-dwelling older adults.
iii. *Optimizing HDL-C levels through pedometer-based walking: a long-term perspective:*
  Bjorgaas et al., [72] studied Pedometer/no-pedometer groups and found no significant between-group differences in triglycerides and HDL-C. However, the study reported that the participants’ steps per day did not increase from study month 1 to 6 (*p* = .65) in the pedometer group, and no specific number of steps per day was set as the target for the participants. Invariably, a lack of increase in step count implies that the intervention either did not alter the intensity or remained the same. Consequently, no meaningful change in the response variable - HDL-C - should be anticipated. This perspective is validated by the findings from another study [131], in which older adult participants added approximately 1500 steps per day to achieve a daily step count exceeding 10,000 steps throughout the study. In contrast, the study [72] included in our review did not add any steps over the trial's duration. Notably, the participants in the other study [131] demonstrated a significant increase in HDL-C levels after a 59-week pedometer-based walking program. However, no difference in HDL-C was reported at week 21, suggesting that the metabolic impact of a pedometer-based walking program on HDL-C is optimized with long-term (> 1 year) accumulation of physical activity behavior. This could be protective against CVD risks in older adults. Furthermore, it suggests that setting specific targets or thresholds of > 10,000 steps/day for older adults could be more beneficial than asking them to arbitrarily “increase daily step count from one visit to the next.” A study [132] comparing diabetic patients with a pedometer-based walking intervention against a control group found no significant short- or intermediate-term changes in health outcomes (including systolic blood pressure, waist circumference, body mass index, glucose control and fasting glucose), triglycerides, total HDL-C and LDL-C, and steps/day) over 24 weeks.
iv. *Pedometer-Based Walking and Glycemic Control: Unveiling the Threshold:*
  Bjorgaas et al. [72] found no significant change in glycated hemoglobin and fasting blood glucose compared to control. However, within-group comparisons revealed a significant improvement in the pedometer group. Interestingly, the study identified ≥ 4000 steps/day as a threshold beyond which glycated hemoglobin improved significantly in those who attained it, but it did not affect other health outcomes.
v. *Optimizing metabolic health: decoding pedometer-based walking intensity and duration*
  Comparing all the studies above, it appears that metabolic health outcomes respond differently to physical activity intensity and duration when subjected to pedometer-based walking. As the intensity progresses towards > 10,000 steps and extends beyond 1 year, the likelihood of positive health benefits increases. In the current review, seven out of eight included studies [70–76] did not specify any target step counts per day for the participants. However, the only study that set a target of 5,400 - 7,900 steps/day in a group compared its effect with a self-selected intensity group over a 3-month duration. Interestingly, both groups showed increased daily step counts (which did not differ significantly), as well as reduced body mass index, waist circumference, and blood pressure. This implies that pedometer prescription below 10,000 steps/day over a period of less than 1 year could have a similar effect on metabolic outcomes as a self-selected intensity approach. It reinforces the argument that pedometer-based walking programs could be more effective in altering metabolic outcomes when prescribed as recommended above.
vi. *Optimizing metabolic health: decoding pedometer-based walking intensity and duration*
  The reviewed studies exhibit notable methodological differences, which directly impact the observed heterogeneity and the diverse effects of the intervention. Consequently, it is crucial to interpret the findings of each study within a specific context, considering both methodological and clinical approaches. However, it is essential to acknowledge that the number of included studies for each of the identified conditions within metabolic syndrome remains limited. As such, the available evidence is insufficient to form a valid and comprehensive scientific opinion. Further research and larger-scale studies are warranted to enhance our understanding in this area.

### Publication bias assessment

The application of funnel-plot asymmetry tests to detect publication bias is deemed inappropriate or not meaningful in this review as the criteria for applying asymmetry tests were not met [133]. Specifically:

- *Lack of significant heterogeneity*: There was no significant heterogeneity observed across the studies.
- *I* ^*2*^ *Value Below 50%:* The I^2^ value, which quantifies heterogeneity, remained below 50 % in only two (of the eight reviewed studies) instead of 10 studies. Additionally, these studies exhibited statistically significant results in at least one aspect.
- *Maximal-to-Minimal Variance Ratio:* The ratio of the maximal to minimal variance across studies did not exceed 4.

Therefore, based on these considerations, the application of funnel-plot asymmetry tests does not provide meaningful insights in this context [133].

### Quality of evidence

In our assessment, several factors influenced the quality of evidence:

1. *Performance Bias: *In 87.5% of the studies, exercise supervisors were not blinded to the exercise intervention. Additionally, in 75% of the studies, participants were not blinded. This lack of blinding introduces a high risk of performance bias.
2. *Assessor Bias: *Approximately 62.5% of the studies blinded outcome assessors to the intervention. However, this still indicates a high risk of assessor bias among the minority of assessors. A smaller number of assessors could potentially influence outcomes in favor of the intervention group.
3. *Attrition Bias: *There was a low risk of attrition bias. This was due to a moderate dropout rate of greater than 15% in only two (or 25%) of the included studies. However, an intention-to-treat analysis was not performed in these cases.
4. *Evidence Grading: *Evidence from four studies received a level 1 grading, while evidence from the remaining four studies was graded as level 2.
5. *Overall Assessment: *The average study rating across the review was 5.65 (approximately 6), indicating a barely level 1 GRADE rating for evidence quality.

Overall, these assessments contribute to our understanding of the robustness and reliability of the evidence presented in the review.

## Limitations

While our systematic review provides valuable insights into pedometer-based walking interventions among various participant groups, several limitations should be acknowledged:

1. *Heterogeneity:* The inclusion of diverse participant groups, such as sedentary retirees, individuals with type 2 diabetes, hypertension, and cardiac conditions, introduces heterogeneity. This variation in health status, risk factors, and baseline characteristics may impact the overall study results [134].
2. *Baseline Differences:* Participants with risk factors for stroke may exhibit different baseline health profiles, lifestyles, and responses to interventions compared to healthier individuals. These differences could potentially affect the interpretation of outcomes [135].
3. *Outcome Measures:* The outcomes assessed in our systematic review (e.g., physical activity levels, walking frequency, dependency) may be influenced differently by risk factors. For instance: Sedentary retirees might respond differently to pedometer-based or exercise interventions than those with existing health conditions. Similarly, individuals with hypertension or cardiac conditions may have specific exercise limitations or safety concerns.
4. *Generalizability*: Including diverse participant groups impacts the generalizability of our findings [136]. Thus, results from studies involving only sedentary retirees may not directly apply to those with specific health conditions.
5. *Risk of Confounding*: Combining data from different participant groups introduces the risk of confounding factors [136]. Factors such as age and comorbidities may impact the observed effects. Adjusting for these confounders becomes crucial in interpreting the results.
6. *Publication Bias*: Studies involving healthier participants may be more likely to be published, potentially leading to publication bias [137]. This limitation is acknowledged and future research is encouraged to address this bias.

Overall, while our systematic review contributes valuable evidence, researchers should consider these limitations when interpreting the implications for practice and policy.

### Supplementary Information

Supplementary Material 1.

Supplementary Material 2.

## Data Availability

The datasets supporting the conclusions of this article are available in the institutional University of Nigeria repository and will be made easily available on request when required. All requests for the study data should be addressed to the corresponding author via email: sam.ibeneme@unn.edu.ng.

## Abbreviations

WHO: World Health Organisation
LMICs: Lower-Middle income countries
HBA1c: Glycated haemoglobin
INPLASY: International platform of Registered Systematic
.: Reviews and Meta-Analysis Protocols
RCTs: Randomized control trials
CGMs: Continuous glucose monitors
TIAs: Transient ischemic attacks
ECG: Electrocardiogram
Exs: Exercise
CT: Computed Tomography
MRI: Magnetic resonance imaging
MeSH: Medical Subject Heading
NIHR: National Institute of Health Research
PRISMA: Preferred Reporting items for systematic
PRISMA: Reviews and Meta-analysis protocols
PEDro: Physiotherapy Evidence Database
PA: Physical activity
BMI: Body mass index
HDL-C: High-density lipoprotein cholesterol
PAL: Physical activity level
SMD: Standardized mean difference
SBP: Systolic blood pressure
DBP: Diastolic blood pressure
WC: Waist circumference
DALYs: Disability-adjusted life years
CDC: Centers for Disease Control and Prevention
ASCM: American College of Sports Medicine
METs: Metabolic equivalents
SDG: Sustainable Development Goal
