# Supplementary material for: Effectiveness of pedometer-based walking programmes in improving some modifiable risk factors of stroke among community-dwelling older adults: a systematic review, theoretical synthesis and meta-analysis
Source: BMC Geriatr. 2024 Jun 13;24:516. doi: 10.1186/s12877-024-05069-z (PMC11177376; doi:10.1186/s12877-024-05069-z)
Supplement: Supplementary file 1 — Supplementary Material 1. [file 12877_2024_5069_MOESM1_ESM.docx]

**Appendix I: Search terms**

Concept Search terms

Population MeSH terms: community dwelling elderly population

Free text terms: older adults, geriatrics, aged.

AIntervention MeSH terms: Pedometer based walking programme

Free text terms: Pedometer based walking, physical walking, exertion, home based walking programme

Comparator MeSH terms: clinical trials

Free text terms: Clinical trial, Random allocation, randomly, randomized, trial

Outcomes MeSH terms: modifiable risk factors of stroke

Free text terms: Obesity, hypertension, diabetes, physical activity, heart disease, high cholesterol

In addition to the electronic database search, published systematic reviews of exercise interventions; reference lists of relevant articles and books; the Cochrane systematic review database; the National Institute of Health Research (NIHR) portfolio for recently completed or ongoing studies; and the current controlled trials register were searched to identify relevant clinical trials.
